# Periacetabular osteotomy versus hip arthroscopy in patients with borderline developmental dysplasia of the hip: A systematic review and multi‐level meta‐analysis

**DOI:** 10.1002/jeo2.70311

**Published:** 2025-07-02

**Authors:** Nikolai Ramadanov, Maximilian Voss, Robert Hable, Robert Prill, Dobromir Dimitrov, Roland Becker, Ingo J. Banke, Marco Haertlé, Sufian S. Ahmad

**Affiliations:** ^1^ Center of Orthopaedics and Traumatology, Brandenburg Medical School University Hospital Brandenburg an der Havel Brandenburg an der Havel Germany; ^2^ Faculty of Health Science Brandenburg Brandenburg Medical School Theodor Fontane Brandenburg an der Havel Germany; ^3^ Faculty of Applied Computer Science Deggendorf Institute of Technology Deggendorf Germany; ^4^ Department of Surgical Propedeutics, Faculty of Medicine Medical University of Pleven Pleven Bulgaria; ^5^ Clinic of Orthopedics and Sports Orthopedics, School of Medicine and Health & Klinikum rechts der Isar Technical University Munich Munich Germany; ^6^ Department of Orthopaedic Surgery Hannover Medical School Hannover Germany

**Keywords:** borderline, dysplasia, meta‐analysis, periacetabular osteotomy, systematic review

## Abstract

**Purpose:**

A comprehensive meta‐analysis is required to address the lack of quantitative evidence on treatment outcomes in borderline developmental dysplasia of the hip (BDDH). This study compares periacetabular osteotomy (PAO) and hip arthroscopy (HAS) through a multi‐level meta‐analysis, providing quantitative insights into their efficacy and safety.

**Methods:**

A systematic literature search was conducted in PubMed, Epistemonikos, and Embase up to February 28, 2025. A frequentist meta‐analysis was performed using the Hartung‐Knapp‐Sidik‐Jonkman heterogeneity estimator. Continuous variables were analyzed using mean values with 95% confidence intervals (CIs), and binary outcomes as proportions with 95% CIs. Sensitivity analysis compared studies defining BDDH as 20–25° versus all included studies. Statistical heterogeneity was assessed using Higgins' *I* ^2^. A random‐effects model was applied in cases of significant heterogeneity.

**Results:**

The literature search identified 39 primary studies with a total of 2075 patients (2121 hips). The test for subgroup differences showed no statistically significant difference between the PAO group and the HAS group in post‐operative mHHS (*χ* ^2^ = 0.55; df = 1; *p* = 0.46), in post‐operative iHOT‐12 (*χ* ^2^ = 0.00; df = 1; *p* = 0.98), in the change in mHHS (*χ* ^2^ = 0.37; df = 1; *p* = 0.54), in the change in iHOT‐12 (*χ* ^2^ = 1.05; df = 1; *p* = 0.30), in MCID of post‐operative functional outcome scores (*χ* ^2^ = 0.43; df = 1; *p* = 0.51), in reoperation (*χ* ^2^ = 0.17; df = 1; *p* = 0.68), and in complications (*χ* ^2^ = 3.35; df = 1; *p* = 0.07). The absolute mean values for nearly all parameters favour PAO, which may indicate a potential advantage.

**Conclusion:**

PAO and HAS yield comparable short‐ to mid‐term outcomes in BDDH. Long‐term studies are needed to determine whether HAS is a definitive treatment or delays structural correction. Future research should standardize BDDH definitions (LCEA 20–25°) to enhance comparability and treatment consistency.

**Level of Evidence:**

A meta‐analysis of retrospective and prospective primary studies.

**Abbreviations**
BDDH: borderline developmental dysplasia of the hip
CI: confidence interval
DDH: developmental dysplasia of the hip
HAS: hip arthroscopy
HHS: Harris Hip Score
HOS‐ADL: Hip Outcome Score – Activities of Daily Living
HOS‐SSS: Hip Outcome Score – Sport Subscale
iHOT: International Hip Outcome Tool
LCEA: lateral centre‐edge angle
MCID: minimal clinically important difference
mHHS: modified Harris Hip Score
NAHS: Non‐Arthritic Hip Score
PAO: periacetabular osteotomy
PRISMA: Preferred Reporting Items for Systematic Reviews and Meta‐Analyses
PROM: patient‐reported outcome measure
PROSPERO: International Prospective Register of Systematic Reviews
RoB: risk of bias
ROBINS: Risk Of Bias In Non‐randomized Studies of Interventions
VAS: visual analogue scale
WOMAC: Western Ontario and McMaster Universities Osteoarthritis Index

## INTRODUCTION

Hip instability is a complex condition that causes pain in young adults and may lead to early‐onset osteoarthritis [12]. One of the most common aetiologies is developmental dysplasia of the hip (DDH), characterized by insufficient bony coverage of the femoral head, diagnosed radiographically by a lateral centre‐edge angle (LCEA) below 25° [55]. The standard surgical treatment for symptomatic DDH is periacetabular osteotomy (PAO), which reorients the acetabulum to improve femoral head coverage and joint biomechanics. Long‐term studies show lasting therapeutic benefits with low rates of conversion to total hip arthroplasty (THA) [59].

A milder form of DHH, known as borderline developmental dysplasia of the hip (BDDH), is defined by an LCEA ranging from 18° to 25° [21, 55] or, according to other authors, from 20° to 25° [53]. Hip arthroscopy (HAS) has emerged as an alternative or adjunctive treatment option alongside PAO. Advances in arthroscopic techniques now enable the repair of stabilizing soft tissues such as the labrum, joint capsule, and ligamentum teres [15]. Capsular management, particularly closure or plication, plays a crucial role in maintaining hip stability.

Three systematic reviews evaluated outcomes of HAS and PAO for BDDH. Barton et al. [3] focused on hip arthroscopy outcomes in 505 patients, showing meaningful patient‐reported outcome measures (PROMs) improvement but a high reoperation rate (13.7%). Murata et al. [38] examined 12 studies and reported improved outcomes for both interventions but highlighted the lack of consensus on the optimal treatment approach. Kuhns et al. [27] analyzed 26 studies, finding significant post‐operative improvement in PROMs for both procedures, though higher alpha angles in HAS studies. A key limitation across all systematic reviews is the absence of a solid formal meta‐analysis, preventing pooled data synthesis and statistical comparisons. Importantly, none of the reviews accounted for variability in LCEA definitions or stratified results by LCEA subgroups, limiting applicability to borderline dysplasia. Additionally, high heterogeneity in study populations, surgical techniques, and outcome measures further limits comparability. Inadequate treatment of BDDH may result in progression to early osteoarthritis or necessitate revision surgery, underscoring the clinical importance of accurate diagnosis and tailored intervention. More robust, high‐level evidence is needed to determine the superior treatment strategy for BDDH.

A reliable meta‐analysis is needed to address the existing gap in orthopaedic literature regarding BDDH patient outcomes. The aim of this study was to conduct a multi‐level meta‐analysis to systematically compare the outcomes of PAO and HAS in the treatment of BDDH, providing quantitative insights into their relative efficacy and safety.

## METHODS

### Reporting guidelines and protocol registration

The study protocol was registered in the International Prospective Register of Systematic Reviews (PROSPERO) on 17 February 2025. The updated Preferred Reporting Items for Systematic Reviews and Meta‐Analyses (PRISMA) guidelines [44] were adhered to rigorously. The corresponding PRISMA checklist, outlining key reporting criteria, is available in the Supporting Information.

### Data sources and search strategies

A systematic literature search was performed across several databases, including PubMed, Epistemonikos, and Embase, covering publications up to 28 February 2025. The search employed a Boolean strategy to identify relevant primary studies investigating PAO or HAS procedures in BDDH patients. The query was adapted for each database's specific search syntax: (((borderline dysplasia) OR (BDDH) OR (BHD)) AND ((arthroscopy) OR (periacetabular osteotomy) OR (PAO))). There were no limitations on publication year or language.

### Study screening and selection

A two‐stage screening process was implemented. Initially, two independent reviewers (NR and MV) assessed study titles and abstracts for relevance. Full‐text versions of potentially eligible studies were then retrieved and re‐evaluated by both reviewers to determine their inclusion in the final meta‐analysis. Consensus between the two reviewers (NR and MV) guided study selection, with any disagreements resolved through discussion with a third reviewer (SA). The kappa coefficient (*κ*) was calculated to measure inter‐reviewer agreement.

### Inclusion and exclusion criteria

The present meta‐analysis considered randomized controlled trials (RCTs) and non‐RCTs, including both prospective and retrospective studies, as well as case series. Excluded from consideration were case reports, editorials, and review articles. The focus was on two main interventions for BDDH patients: PAO and HAS procedures. Studies that did not report outcomes pertinent to the research question were excluded.

### Types of outcome measures

Hip function was assessed using multiple PROMs, including the Harris Hip Score (HHS) or the modified HHS (mHHS), the Western Ontario and McMaster Universities Osteoarthritis Index (WOMAC), Non‐Arthritic Hip Score (NAHS), International Hip Outcome Tool (iHOT), the Hip Outcome Score – Activities of Daily Living (HOS‐ADL) and Hip Outcome Score – Sport Subscale (HOS‐SSS). Pain was assessed using the visual analogue scale (VAS).

### Data extraction and analysis

Data extraction was carried out independently by two reviewers (NR and MV). Any inconsistencies were resolved through discussion with a third reviewer (SA). Extracted information included first author, publication year, study origin, sample size, patient characteristics, study design, risk of bias, key outcome measures, and follow‐up duration. In cases of missing outcome data, corresponding authors were contacted for clarification. If no response was received, missing standard deviations were imputed using established methods. Only PROMs validated for HAS and PAO were included in the meta‐analysis. PROM scores were converted into minimal clinically important difference (MCID) [43] units by normalizing against the most conservative MCID reported in the literature [23, 42, 46, 52] (Table 1). For studies reporting multiple PROMs, the prioritization followed this sequence: mHHS, WOMAC, NAHS, iHOT‐12, HOS‐ADL and HOS‐SSS. Given the variability in PROMs across studies, we prioritized the most commonly and consistently reported measures. As no formal guideline exists, this approach was chosen to enhance comparability and enable meaningful data synthesis. To ensure reliable and comprehensive results, we calculated the differences between post‐operative and preoperative PROM scores. Furthermore, the incidence of reoperation and complications, including infection, deep vein thrombosis, pulmonary embolism, nerve injury, loss of reduction, nonunion and heterotopic ossification, was also extracted.

**Table 1:** PROMs with their corresponding MCIDs.

PROM	MCID unit
mHHS	8.20 [42]
NAHS	10.00 [46]
iHOT‐12	9.00 [52]
HOS‐ADL	9.00 [23]
HOS‐SSS	14.50 [42]

Abbreviations: HOS‐ADL, Hip Outcome Score – Activities of Daily Living; HOS‐SSS, Hip Outcome Score ‐ Sports Subscale; iHOT, International Hip Outcome Tool; MCID, minimal clinically important difference; mHHS, modified Harris Hip Score; NAHS, Non‐Arthritic Hip Score; PROM, patient‐reported outcome measure.

### Categories of outcome measures

Three categories of outcome measures were meta‐analyzed to provide a comprehensive comparison between groups: (i) post‐operative functional scores (mHHS, WOMAC, NAHS, iHOT‐12, HOS‐ADL and HOS‐SSS) and post‐operative pain score (VAS). (ii) The differences between post‐operative and preoperative scores (change in functional outcome). (iii) The MCID of the post‐operative functional scores. (iv) Incidence of reoperation and complications.

### Quality assessment

Two independent reviewers (NR and MV) conducted quality assessments of the included studies. Risk of bias (RoB) was assessed using the Risk Of Bias In Non‐randomized Studies of Interventions (ROBINS‐I) tool [50] for non‐randomized studies. Any disagreements were settled through discussion with a third reviewer (SA). The kappa coefficient (*κ*) was calculated to measure inter‐reviewer agreement. Furthermore, publication bias was assessed using Begg's test or represented through funnel plots.

### Measures of treatment effect

A frequentist meta‐analysis was conducted where differences between PAO and HAS procedures were evaluated via subgroup analyses. The Hartung–Knapp–Sidik–Jonkman heterogeneity estimator was applied [24, 45]. Functional and pain outcomes, being continuous variables, were assessed using mean values along with 95% confidence intervals (CIs). Reoperation and complication incidence, being binary outcomes, were assessed using rates of occurrence (proportions) with 95% CIs. A sensitivity analysis [39] was performed to compare studies that defined BDDH as 20–25° to all included studies. Statistical heterogeneity was assessed using the Higgins *I* ^2^ statistic, with the following categories: low (<25%), moderate (25%–75%) and high (>75%). If significant heterogeneity was detected, a random‐effects model was used instead of a fixed‐effects model. All analyses were conducted by a qualified statistician (RH) using the R packages meta and metafor [47].

## RESULTS

### Systematic review

**Figure 1:**
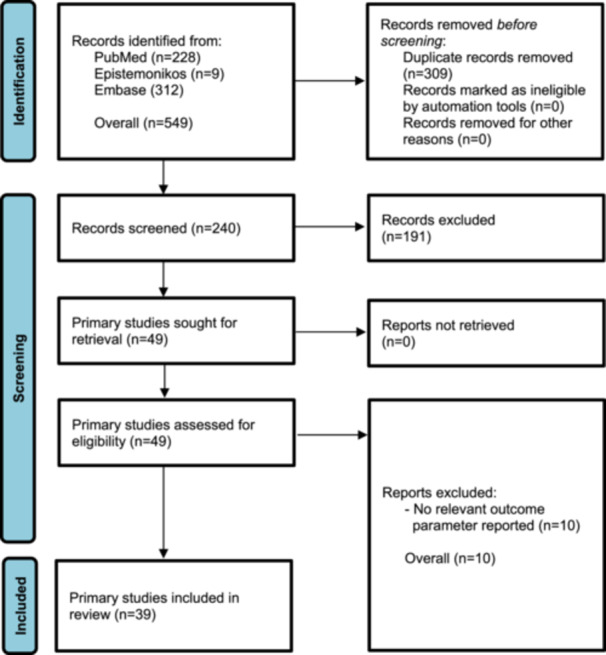
Flow chart diagram.

In the systematic review of PubMed, Epistemonikos and Embase, a total of 240 records were screened for title and abstract with high inter‐reviewer agreement (*κ* = 0.98) after removal of 309 duplicates. A total of 49 primary studies were assessed for eligibility with full inter‐reviewer agreement (*κ* = 1.0), whereas 10 primary studies were excluded because they did not report an outcome of interest. Ultimately, the systematic review of the literature identified 39 primary studies [1, 2, 4, 5, 6, 7, 8, 9, 10, 11, 13, 14, 16, 17, 18, 19, 20, 22, 25, 26, 28, 29, 30, 31, 32, 33, 34, 35, 36, 37, 40, 41, 48, 49, 51, 54, 56, 57, 58] with a total of 2075 patients (2121 hips) that met the eligibility criteria for inclusion in the meta‐analysis (Figure 1).

### Characteristics of the included primary studies

The key characteristics of the 39 primary studies included in this meta‐analysis are summarized in Table 2. The PAO group consisted of 534 patients (550 hips) from 8 primary studies [2, 18, 22, 28, 36, 37, 41, 49] and the HAS group consisted of 1541 patients (1571 hips) from 31 primary studies [1, 4, 5, 6, 7, 8, 9, 10, 11, 13, 14, 16, 17, 19, 20, 25, 26, 29, 30, 31, 32, 33, 34, 35, 40, 48, 51, 54, 56, 57, 58]. The mean age of patients in the PAO group was 28.5 years (range: 25 to 33.6), with 86.1% of the patients being women. The mean age of patients in the HAS group was 30.8 years (range: 15.5–39.2), with 66.3% of the patients being women. The mean follow‐up duration was 55.7 months in the PAO group and 49.1 months in the HAS group. The mean mHHS score was 59.8 points for the PAO group and 60.7 points for the HAS group.

**Table 2:** Characteristics of the patient cohort.

Primary study	Year of publication	Origin	Study design	LCEA	Hips	Patients	Operation	Patient age	Female sex	Follow‐up	Preoperative mHHS
				(in °)	*N*	*N*		in years; mean ± SD (range)	*N* (%)	in months; mean ± SD (range)	in points; mean ± SD
Alter et al. 2021 [1]	2021	USA	Retrospective case series	20–25	41	41	HAS	29.6 ± 13.4	31 (75.6)	26 ± 5	61.2 ± 11.9
							– Chondral lesion debridement – Labral repair				
Andronic et al. 2023 [2]	2023	Switzerland	Retrospective case series	18–25	42	40	PAO	27.0 (15.0–40.0)	28 (70.0)	96 (67–139)	73.0 ± 10.5
Beals et al. 2021 [4]	2021	USA	Retrospective case series	20–25	38	38	HAS		23 (60.5)	120	58.0 ± 15.0
							– Chondral lesion debridement – Femoral osteochondroplasty – Labral repair				
Beck et al. 2019 [5]	2019	USA	Retrospective cohort study	20–25	112	112	HAS	33.6 ± 12.7		24	55.6 ± 14.5
							– Acetabuloplasty – Labral repair				
Chaharbakhshi et al. 2017 [6]	2017	USA	Prospective cohort study	18–25	40	40	HAS	28.2 (13.3–51.9)	36 (90.0)	46 (24–83)	65.5
							– Acetabuloplasty – Chondral lesion debridement – Labral repair – Microfracture				
Chandrasekaran et al. 2017 [7]	2017	USA	Retrospective case series	18–25	55	55	HAS	24.3 (13.2–38.7)	35 (63.6)	25.2	63.7
							– Capsular plication – Chondral lesion debridement – Femoral osteochondroplasty – Labral repair – Microfracture				
Chapman et al. 2024 [8]	2024	USA	Retrospective cohort study	20–25	28	28	HAS		20 (71.4)	120	53.1 ± 13.9
							– Chondral lesion debridement – Femoral osteochondro‐plasty – Labral repair				
Cvetanovich et al. 2017 [9]	2017	USA	Retrospective cohort study	18–25	36	36	HAS	31.5 ± 11.8	27 (75.0)	≥24	57.2 ± 12.3
							– Acetabuloplasty – Chondral lesion debridement – Labral repair				
D'Ambrosi et al. 2021 [10]	2021	Italy	Retrospective case series	18–25	25	25	HAS	33.6 ± 8.6	20 (80.0)	54 ± 23	
							– Chondral lesion debridement – Femoral osteochondroplasty – Labral repair				
Domb et al. 2013 [11]	2013	USA	Retrospective case series	15‐25	22	22	HAS	20.0 (14.0–39.0)	18 (82.0)	28 (17–39)	69.0
							– Acetabuloplasty – Chondral lesion debridement – Femoral osteochondroplasty – Labral repair				
Domb et al. 2018 [13]	2018	Usa	Retrospective case series	18–24	21	19	HAS	22.9 (15.5–39.3)	19 (90.0)	69 (60–84)	70.3 ± 9.8
							– Acetabuloplasty – Capsular plication – hondral lesion debridement – Femoral osteochondroplasty – Labral repair				
Domb et al. 2023 [14]	2023	USA	Prospective cohort study	18–25	45	45	HAS	31. ± 12.9 (14.9–62.2)	38 (84.4)	120	66.6 ± 15.3
							– Acetabuloplasty – Capsular plication – Chondral lesion debridement – Femoral osteochondroplasty – Labral repair – Microfracture				
Evans et al. 2017 [16]	2017	USA	Retrospective case series	20–25	21	21	HAS	15.5 (13.1–17.5)	17 (81.0)	26	59.7
							– Chondral lesion debridement – Labral repair				
Feghhi et al. 2020 [17]	2020	USA	Retrospective cohort study	18–24	19	18	HAS	28.0 (15.0–52.0)	14 (77.8)	44	64.7
							– Capsular plication – Capsular repair – Chondral lesion debridement – Femoral osteochondroplasty – Labral repair – Microfracture				
Fischer et al. 2024 [18]	2024	Germany	Prospective case series	18–25	94	91	PAO	31.0 ± 8.2	74 (81.3)	28 ± 11	52.0 ± 17.0
Foissey et al. 2023 [19]	2023	France	Retrospective case series	18–25	39	37	HAS	31.0 ± 10.0 (15.0–47.0)	12 (31.0)	34 ± 1 (24‐57)	55.0 ± 14.0
							– Capsular plication – Labral repair				
Fukui et al. 2015 [20]	2015	USA	Retrospective case series	20–25	102	102	HAS	35.0 (18.0–69.0)	51 (50.0)	40 (24–97)	63.5 ± 14.0
							– Acetabuloplasty – Capsular plication – Chondral lesion debridement – Labral repair – Microfracture				
Grammatopoulos et al. 2018 [22]	2018	England/Canada/USA	Prospective cohort study	15–25	61	61	PAO	25.0 ± 9.0 (14.0–52.0)	51 (83.6)	48 ± 18 (24–102)	60.0 ± 13.0
Hatakeyama et al. 2018 [25]	2018	Japan	Retrospective cohort study	20–25	45	45	HAS	31.4 (12.0–65.0)	30 (66.7)	42 (24–73)	71.1 ± 20.9
							– Acetabuloplasty – Capsular plication – Femoral osteochondro‐ plasty – Labral repair				
Kalore and Jiranek 2012 [26]	2012	USA	Retrospective cohort study	23	50	50	HAS	38.0	40 (88.0)	33 (5–100)	53.9
							– Acetabuloplasty – Chondral lesion debridement – Labral repair				
Leopold et al. 2024 [28]	2024	Germany	Retrospective case series	18–25	55	52	PAO	28.9 ± 8.6	46 (88.5)	62 ± 9	
Li et al. 2024 [29]	2024	USA	Retrospective cohort study	20–25	31	31	HAS	36.0 ± 12.7	22 (71.0)	24	43.1 ± 11.6
							– Acetabuloplasty – apsular plication – Chondral lesion debridement – Femoral osteochondro‐ plasty – Labral repair – Microfracture				
Liu et al. 2024 [30]	2024	China	Retrospective cohort study	18–25	21	21	HAS	28.6 ± 5.5	13 (61.9)	86 ± 5	57.7 ± 7.4
							– Acetabuloplasty – Femoral osteochondroplasty – Labral repair				
Maldonado et al. 2018 [31]	2018	USA/Israel	Retrospective case control	18‐25	122	115	HAS	24.6 ± 7.6	99 (86.1)	40 ± 18	64.2 ± 15.2
							– Acetabuloplasy – Capsular plication – Condral lesion debridement – Femoral osteochondroplasty – Labral repair – Microfracture				
Marland et al. 2021 [32]	2021	USA	Retrospective cohort study	18–25	249	249	HAS	33.1 ± 11.1 (14.0–59.0)	249 (100.0)	35 (24–48)	
							– NR				
Mas Martinez et al. 2020 [33]	2020	Spain	Retrospective cohort study	20–24	20	20	HAS	37.8 ± 8.9 (18.0–50.0)	11 (55.0)	50 ± 10 (30–60)	75.3 ± 7.3
							– Acetabuloplasty – Capsular plication – Labral repair – Femoral osteochondroplasty – Microfracture				
Matsuda et al. 2019 [34]	2019	USA	Retrospective cohort study	20–25	49	49	HAS	34.6	33 (67.3)	24	
							– NR				
McCarthy et al. 1998 [35]	1998	USA	Retrospective case series	19–27	20	20	HAS	36.0 (16.0–54.0)	13 (65.0)	24 (27–41)	
							– NR				
McClincy et al. 2019 [36]	2019	USA	Retrospective case series	18–25	49	49	PAO	26.5 ± 8.0	46 (93.9)	26 (24–48)	64.0 ± 19.0
Mose et al. 2019 [37]	2019	Sweden/Denmark	Prospective cohort study	20–25	44	44	PAO	33.6 ± 12.6	40 (90.9)	24	
Nawabi et al. 2016 [40]	2016	USA	Prospective cohort study	18–25	55	46	HAS	29.8 ± 9.4	22 (48.0)	16 (4–41)	61.7 ± 10.9
							– Femoral osteochondroplasty – Labral repair				
Nepple et al. 2023 [41]	2023	USA	Retrospective case series	18–25	186	178	PAO	25.2 ± 8.5 (14.0–45.0)	157 (88.2)	39 ± 24	58.0 ± 14.0
Selley et al. 2023 [48]	2023	USA	Retrospective cohort study	18–25	38	33	HAS	29.9 ± 9.3	21 (63.6)	115 (98–139)	62.0 ± 11.6
							– Chondral lesion debridement – Labral repair				
Sierra et al. 2017 [49]	2017	USA	Retrospective cohort study	18–25	19	19	PAO	31.0 (12.0–56.0)	18 (94.7)	121 (24–236)	52.0
Tassinari et al. 2021 [51]	2021	Italy	Retrospective cohort study	18–25	15	15	HAS	31.0 (16.0–39.0)	5 (33.3)	24	
							– Chondral lesion debridement – Femoral osteochondroplasty – Labral repair – Microfracture				
Wang et al. 2021 [54]	2021	China	Retrospective case series	20–25	36	34	HAS	30.9 (12.0–54.0)	27 (79.4)	69 (24–150)	64.5 ± 7.9
							– Femoral osteochondroplasty – Labral repair				
Yang et al. 2023 [56]	2023	China	Retrospective cohort study	20–25	77	77	HAS	36.1 ± 9.8	39 (50.6)	45 ± 8	61.9 ± 11.2
							– Chondral lesion debridement – Labral repair				
Yoon et al. 2019 [57]	2019	South Korea	Retrospective case series	20–25	47	45	HAS	39.2 ± 11.8		26	61.0 ± 7.6
							– Chondral lesion debridement – Femoral osteochondroplasty – Labral repair				
Zhang et al. 2023 [58]	2023	China	Retrospective cohort study	18–25	52	52	HAS	30.8 ± 8.4 (18–49)	37 (71.2)	44 ± 11 (25–64)	39.4 ± 12.3
							– Chondral lesion debridement – Femoral osteochondroplasty – Labral repair – Microfracture				

Abbreviations: HAS, hip arthroscopy; LCEA, lateral centre‐edge angle; mHHS, modified Harris Hip Score; PAO, periacetabular osteotomy; SD, standard deviation.

### Quality assessment

**Figure 2:**
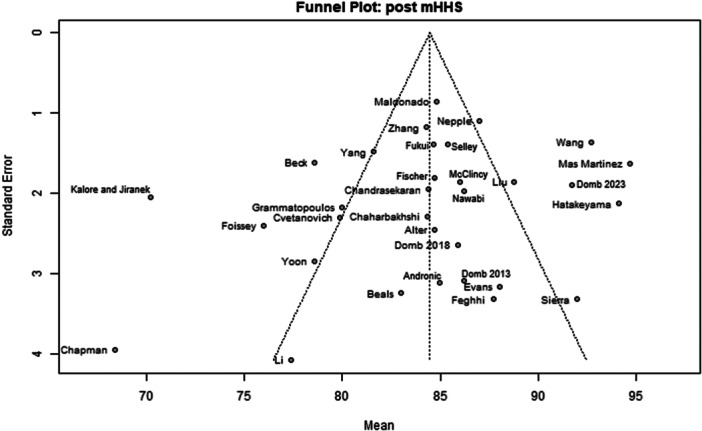
Funnel plot of post‐operative mHHS. mHHS, modified Harris Hip Score.

**Figure 3:**
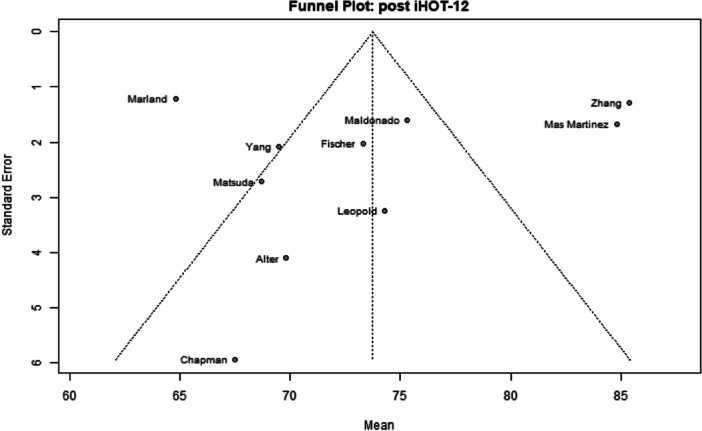
Funnel plot of post‐operative iHOT‐12. iHOT, International Hip Outcome Tool.

**Figure 4:**
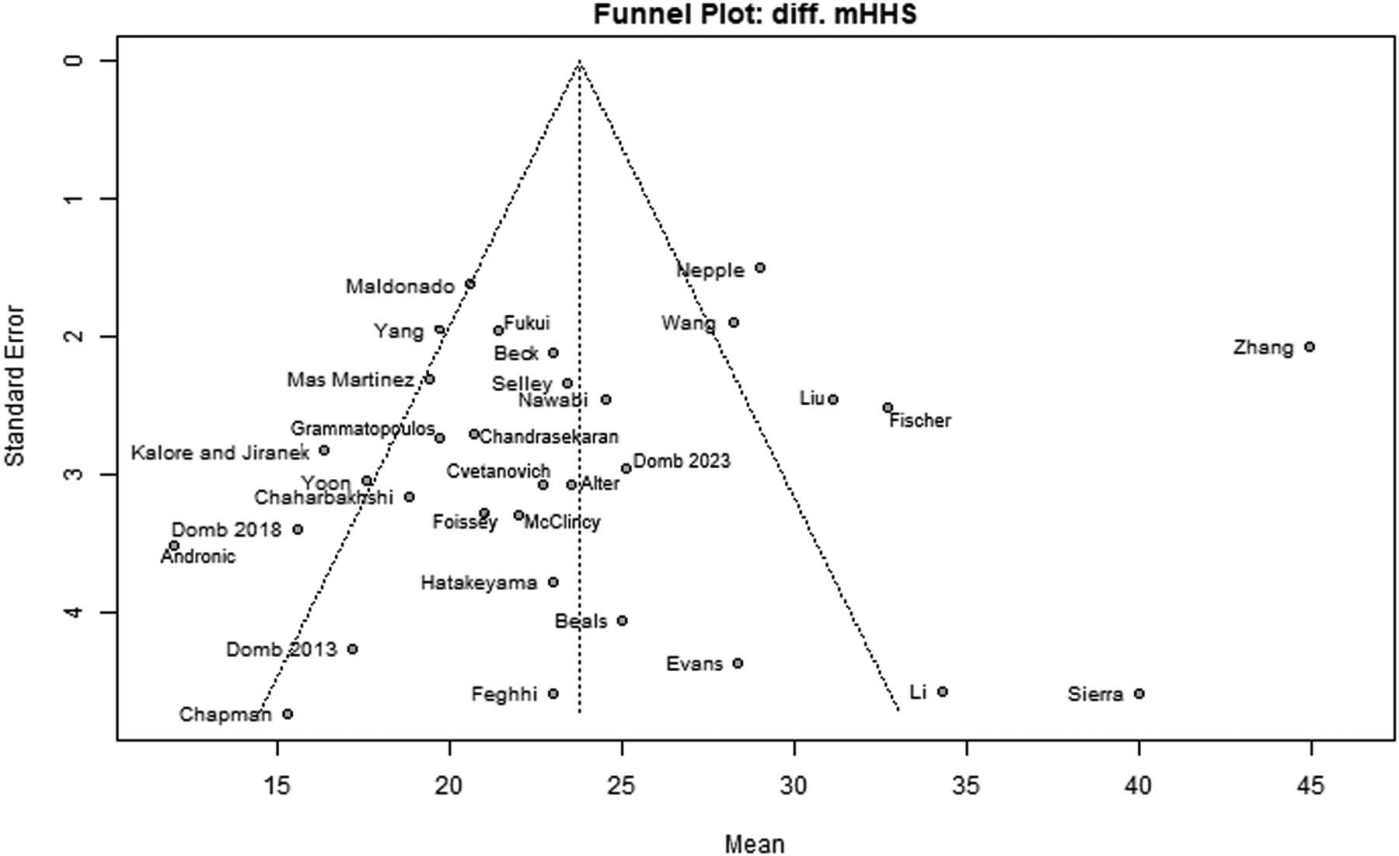
Funnel plot of the change in mHHS. mHHS, modified Harris Hip Score.

**Figure 5:**
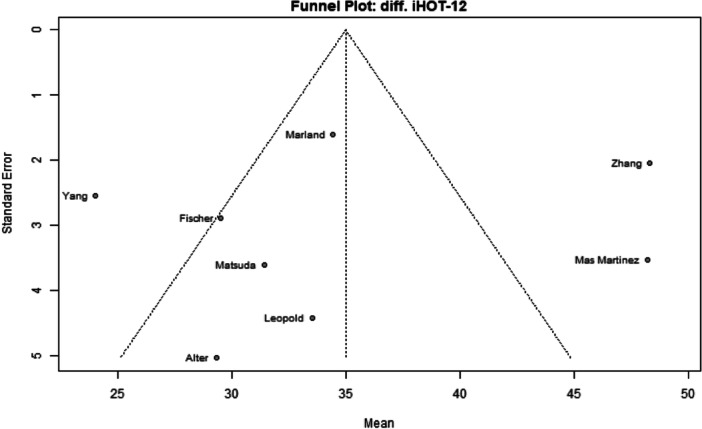
Funnel plot of the change in iHOT‐12. iHOT, International Hip Outcome Tool.

**Figure 6:**
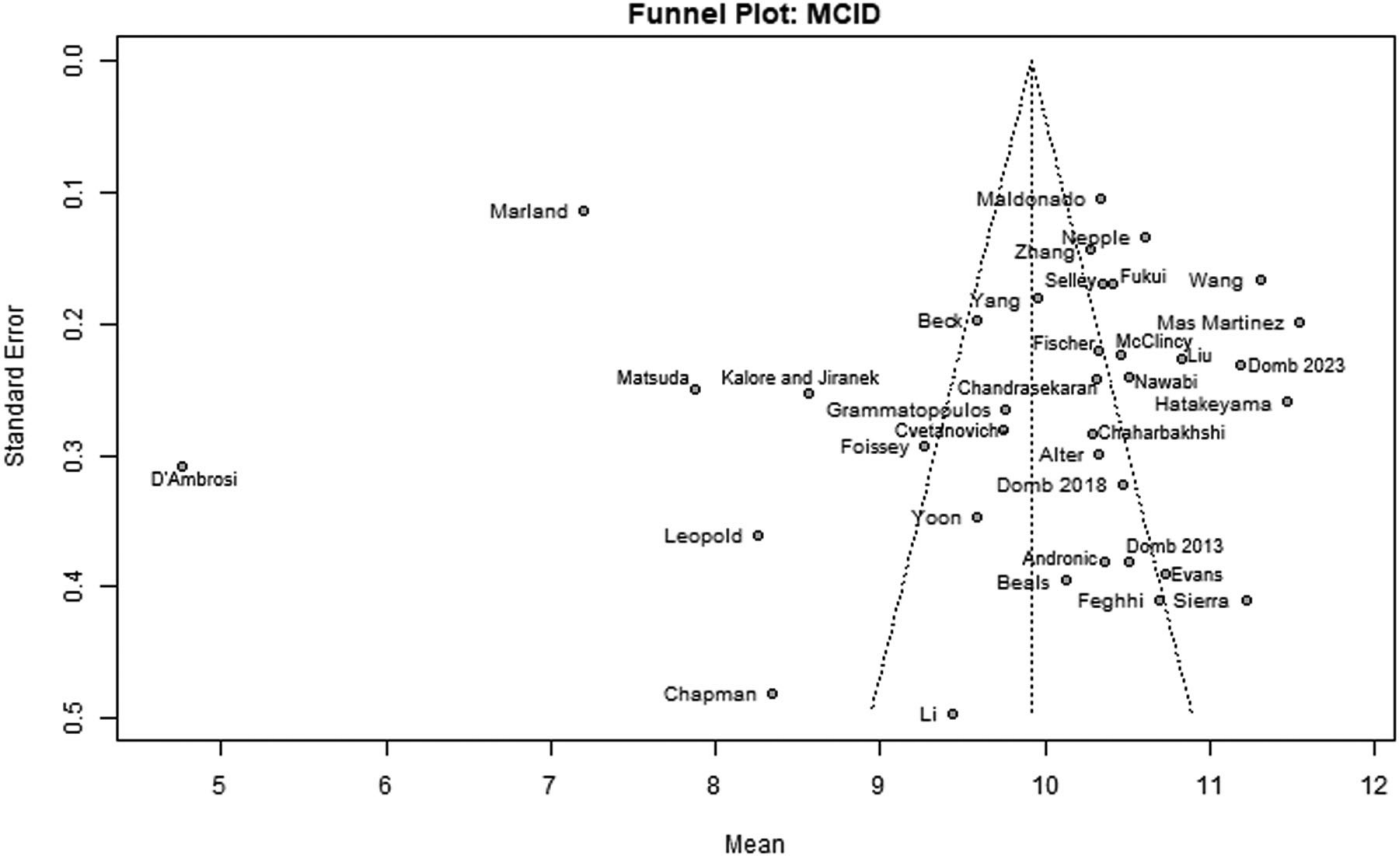
Funnel plot of MCID. MCID, minimal clinically important difference.

**Figure 7:**
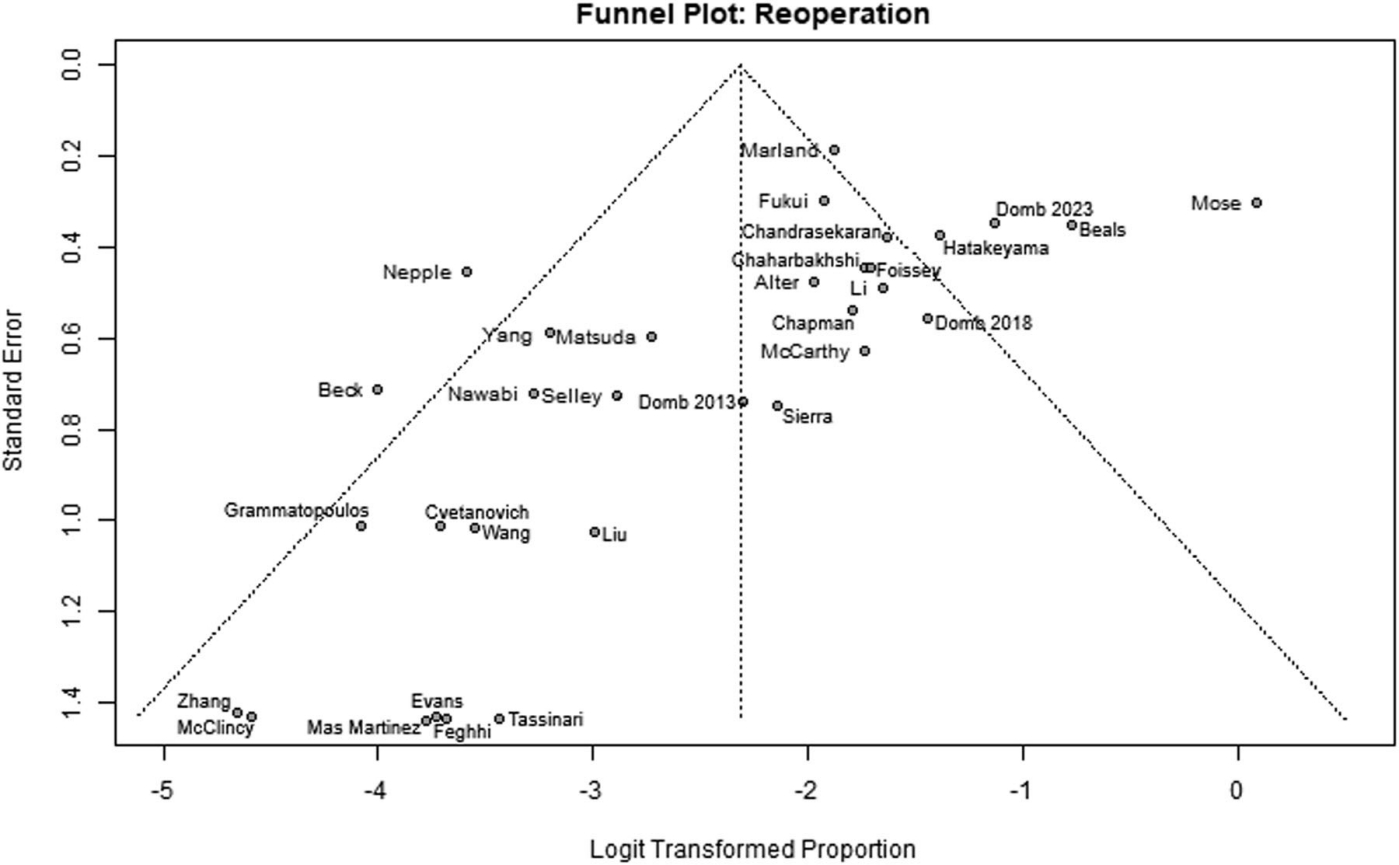
Funnel plot of reoperation.

**Figure 8:**
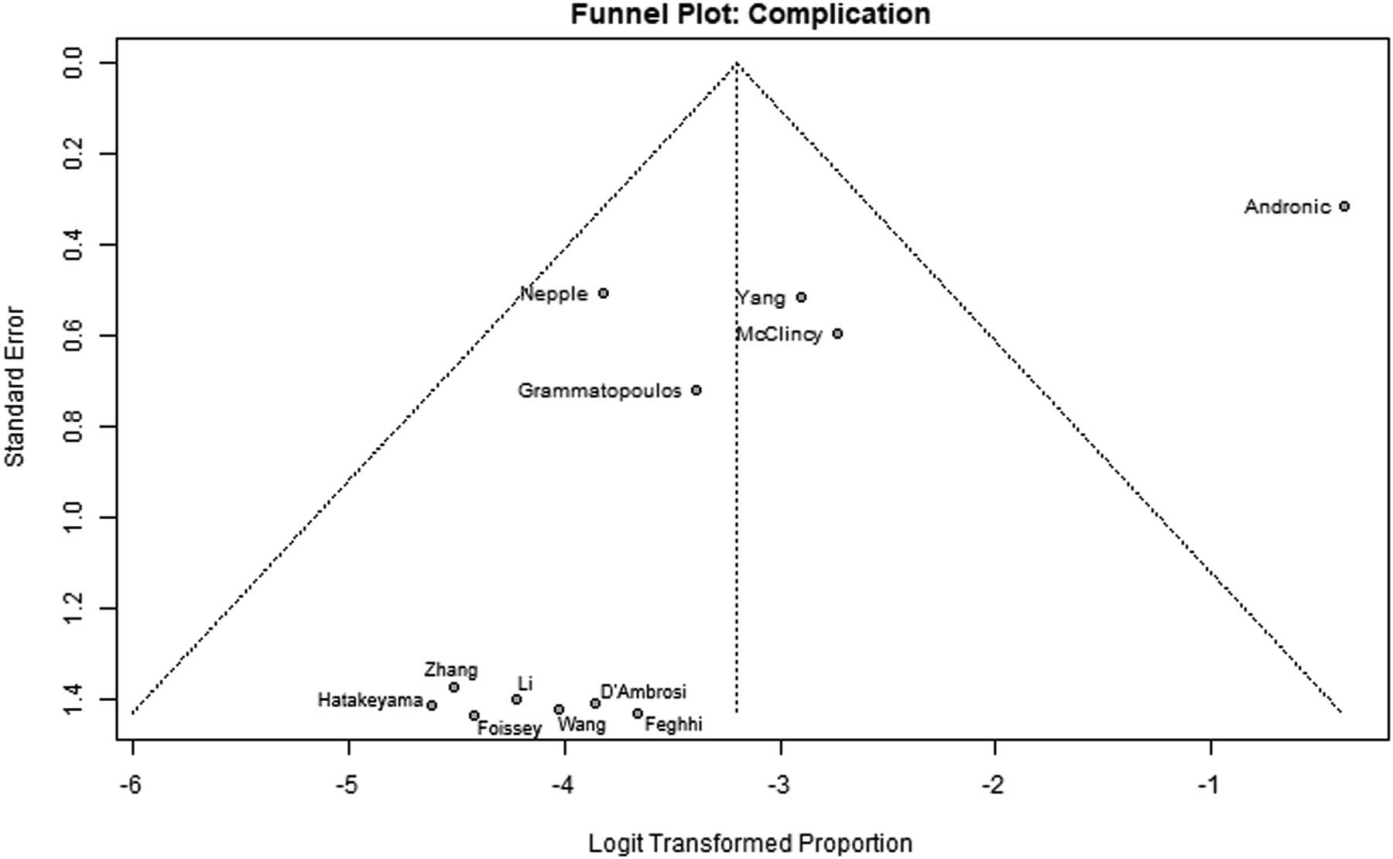
Funnel plot of complications.

Among the 39 primary studies included, 22 were rated with a low risk of bias [4, 6, 7, 10, 11, 13, 14, 18, 19, 22, 29, 33, 35, 36, 37, 40, 41, 51, 54, 56, 57, 58], 17 were rated with a moderate risk of bias [1, 2, 5, 8, 9, 16, 17, 20, 25, 26, 28, 30, 31, 32, 34, 48, 49] (Table 3). The risk of bias assessment demonstrated high inter‐reviewer agreement (Cohen's *κ* = 0.97). The funnel plots (Figures 2, 3, 4, 5, 6, 7, 8) showed a low publication bias for the post‐operative iHOT‐12 and for the change in iHOT‐12 (Figures 3 and 5), a moderate publication bias for post‐operative mHHS, reoperation and complications (Figures 2, 7 and 8), a high publication bias for the change in mHHS, MCID of post‐operative functional outcome scores (Figures 4 and 6).

**Table 3:** Risk of bias assessment.

Author	Pre‐intervention		At intervention	Post‐intervention				Overall risk of bias
	Bias due to confounding	Bias in selection of participants in the study	Bias in classification of interventions	Bias due to deviations from intended interventions	Bias due to missing data	Bias in measurement of outcomes	Bias in selection of the reported result	
Alter et al. 2021 [1]	Low	Moderate	Low	Low	Low	Moderate	Moderate	Moderate
Andronic et al. 2023 [2]	Moderate	Moderate	Low	Low	Low	Moderate	Low	Moderate
Beals et al. 2021 [4]	Moderate	Low	Low	Low	Low	Low	Moderate	Low
Beck et al. 2019 [5]	Low	Moderate	Low	Low	Low	Moderate	Moderate	Moderate
Chaharbakhshi et al. 2017 [6]	Low	Low	Low	Low	Low	Low	Moderate	Low
Chandrasekaran et al. 2017 [7]	Low	Low	Moderate	Low	Low	Low	Low	Low
Chapman et al. 2024 [8]	Moderate	Moderate	Low	Low	Low	Moderate	Moderate	Moderate
Cvetanovich et al. 2017 [9]	Moderate	Low	Low	Low	Low	Moderate	Moderate	Moderate
D'Ambrosi et al. 2021 [10]	Moderate	Low	Low	Low	Low	Low	Moderate	Low
Domb et al. 2013 [11]	Moderate	Low	Low	Low	Low	Low	Moderate	Low
Domb et al. 2018 [13]	Moderate	Low	Low	Low	Low	Low	Moderate	Low
Domb et al. 2023 [14]	Moderate	Low	Low	Low	Low	Low	Moderate	Low
Evans et al. 2017 [16]	Moderate	Low	Low	Low	Low	Moderate	Moderate	Moderate
Feghhi et al. 2020 [17]	Moderate	Moderate	Low	Low	Low	Moderate	Moderate	Moderate
Fische et al. 2024 [18]	Low	Low	Low	Low	Low	Moderate	Moderate	Low
Foissey et al. 2023 [19]	Low	Moderate	Low	Low	Low	Moderate	Low	Low
Fukui et al. 2015 [20]	Moderate	Low	Low	Low	Low	Moderate	Moderate	Moderate
Grammatopoulos et al. 2018 [22]	Low	Low	Low	Low	Low	Low	Moderate	Low
Hatakeyama et al. 2018 [25]	Low	Moderate	Low	Moderate	Low	Moderate	Moderate	Moderate
Kalore and Jiranek 2012 [26]	Low	Moderate	Low	Low	Low	Moderate	Moderate	Moderate
Leopold et al. 2024 [28]	MEDIUM	MEDIUM	Low	Low	Moderate	Moderate	Moderate	Moderate
Li et al. 2024 [29]	Low	Moderate	Low	Low	Low	Low	Moderate	Low
Liu et al. 2024 [30]	Moderate	Moderate	Low	Low	Low	Low	Moderate	Moderate
Maldonado et al. 2018 [31]	Low	Moderate	Low	Low	Moderate	Moderate	Moderate	Moderate
Marland et al. 2021 [32]	Moderate	Moderate	Low	Low	Low	Low	Moderate	Moderate
Mas Martinez et al. 2020 [33]	Low	Moderate	Low	Low	Low	Low	Moderate	Low
Matsuda et al. 2019 [34]	Low	Moderate	Low	Low	Low	Moderate	Moderate	Moderate
McCarthy et al. 1998 [35]	Moderate	Low	Low	Low	Low	Low	Moderate	Low
McClincy et al. 2019 [36]	Moderate	Moderate	Low	Low	Low	Low	Moderate	Low
Mose et al. 2019 [37]	Moderate	Low	Low	Low	Low	Low	Moderate	Low
Nawabi et al. 2016 [40]	Low	Low	Low	Low	Low	Moderate	Moderate	Low
Nepple et al. 2023 [41]	Low	Low	Low	Low	Low	Low	Low	Low
Selley et al. 2023 [48]	Moderate	Moderate	Low	Low	Low	Moderate	Moderate	Moderate
Sierra et al. 2017 [49]	Moderate	Moderate	Low	Low	Low	Low	Moderate	Moderate
Tassinari et al. 2021 [51]	Low	Moderate	Low	Low	Low	Low	Moderate	Low
Wang et al. 2021 [54]	Low	Moderate	Low	Low	Low	Low	Moderate	Low
Yang et al. 2023 [56]	Low	Moderate	Low	Low	Low	Low	Low	Low
Yoon et al. 2019 [57]	Low	Moderate	Low	Low	Low	Low	Moderate	Low
Zhang et al. 2023 [58]	Low	Moderate	Low	Low	Low	Low	Moderate	Low

*Note*: green colour: low risk of bias; yellow colour: moderate risk of bias.

### Meta‐analysis

#### Post‐operative functional outcome

##### Post‐operative mHHS

**Figure 9:**
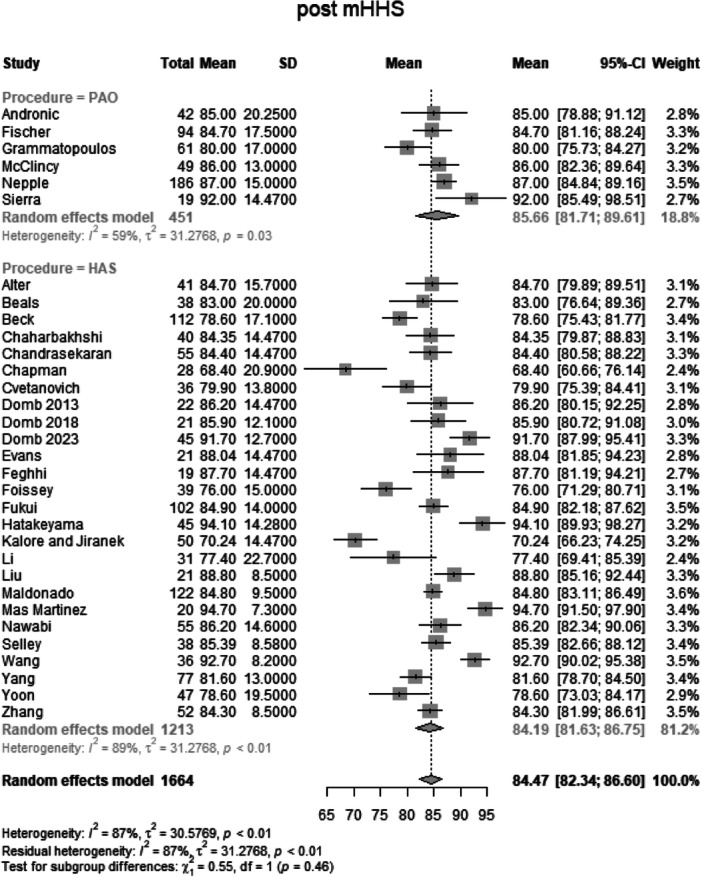
Forest plot of post‐operative mHHS. CI, confidence interval; mHHS, modified Harris Hip Score; SD, standard deviation*.*

Data from 1664 patients from 32 primary studies were pooled (Figure 9, Table 4), with the PAO group consisting of 451 patients and the HAS group consisting of 1213 patients. The mean mHHS of the PAO group was 85.7 points (mean: 85.66, CIs: 81.71–89.61; *I* ^2^ = 59%; *τ* ^2^ = 31.28, *p* = 0.03). The mean mHHS of the HAS group was 84.2 points (mean: 84.19, CIs: 81.63–86.75; *I* ^2^ = 89%; *τ* ^2^ = 31.28, *p* < 0.01). The test for subgroup differences showed no statistically significant difference between the PAO group and the HAS group in post‐operative mHHS (*χ* ^2^ = 0.55; df = 1; *p* = 0.46).

**Table 4:** Summarized results of the meta‐analysis.

	Primary studies, *N*	Patients, *N*	Mean	CIs	*τ* ^2^	*I* ^2^	Heterogeneity *p*	Egger bias	Egger *p*	Difference *p*
Preoperative										
mHHS	32	1664	60.54	57.73 to 63.35	56.56	0.94	<0.01*	−0.14	0.96	0.83
WOMAC	4	199	43.81	12.20 to 75.42	391.75	1.00	<0.01*	3.00	0.94	0.02**
NAHS	14	619	59.80	55.49 to 64.10	50.47	0.92	<0.01*	1.31	0.65	0.03**
iHOT‐12	8	637	38.90	34.72 to 43.08	19.97	0.92	<0.01*	4.13	0.25	0.08
HOS‐ADL	15	685	67.68	65.07 to 70.29	17.52	0.81	<0.01*	−0.89	0.68	N/A
HOS‐SSS	20	938	44.86	41.65 to 48.06	38.89	0.84	<0.01*	1.03	0.63	N/A
VAS	9	476	6.04	5.48 to 6.60	0.43	0.84	<0.01*	−0.64	0.81	N/A
Post‐operative										
mHHS	32	1664	84.47	82.34 to 86.60	30.58	0.87	<0.01*	−1.40	0.30	0.46
WOMAC	4	199	44.92	−20.84 to 110.67	1704.38	1.00	<0.01*	−10.95	0.88	0.13
NAHS	14	619	82.97	77.61 to 88.33	80.93	0.97	<0.01*	−2.49	0.54	<0.01***
iHOT‐12	10	787	73.74	68.61 to 78.87	44.09	0.95	<0.01*	−1.56	0.70	0.98
HOS‐ADL	15	685	89.20	86.69 to 91.7	16.69	0.87	<0.01*	−3.99	0.05**	N/A
HOS‐SSS	20	938	76.64	73.88 to 79.39	25.50	0.68	<0.01*	0.24	0.82	N/A
VAS	9	476	2.17	1.66 to 2.69	0.37	0.74	<0.01*	1.60	0.46	N/A
Change in										
mHHS	32	1664	23.76	21.23 to 26.29	41.12	0.86	<0.01*	−1.51	0.33	0.55
WOMAC	4	199	1.03	−34.79 to 36.85	499.77	1.00	<0.01*	−0.18	0.99	0.29
NAHS	14	619	22.88	18.51 to 27.26	47.91	0.83	<0.01*	0.25	0.93	0.33
iHOT‐12	8	637	34.97	27.46 to 42.49	69.79	0.91	<0.01*	−1.49	0.69	0.30
HOS‐ADL	15	685	21.22	18.21 to 24.24	22.19	0.72	<0.01*	2.43	0.23	N/A
HOS‐SSS	20	938	31.24	27.13 to 35.36	59.61	0.69	<0.01*	1.30	0.27	N/A
VAS	9	476	‐3.84	−4.78 to −2.91	1.25	0.87	<0.01*	0.11	0.97	N/A
MCID										
	36	2042	9.92	9.46 to 10.37	1.72	0.97	<0.01*	0.71	0.79	0.51
Reoperation and complications										
Reoperation	33	1728	0.09	0.06 to 0.13	0.93	0.74	<0.01*	−2.36	<0.01*	0.68
Total complications	14	709	0.04	0.02 to 0.08	0.84	0.80	<0.01*	−2.99	<0.01***	0.07
Infection	11	529	0.02	0.01 to 0.03	0.04	0.00	0.99	−0.39	0.45	0.43
Nerve injury	11	413	0.04	0.01 to 0.10	1.03	0.78	<0.01*	−3.09	<0.01*	0.03**
Heterotopic ossifications	11	397	0.02	0.01 to 0.02	0.01	0.00	1.00	−0.55	0.32	0.06

Abbreviations: CI, confidence interval; HOS‐ADL, Hip Outcome Score – Activities of Daily Living; HOS‐SSS, Hip Outcome Score ‐ Sports Subscale iHOT, International Hip Outcome Tool; mHHS, modified Harris Hip Score; NAHS, Non‐Arthritic Hip Score; VAS, visual analogue score; WOMAC, Western Ontario and McMaster Universities Osteoarthritis Index.

* Statistically significant.

** Very statistically significant.

*** Highly statistically significant.

##### Post‐operative iHOT‐12

**Figure 10:**
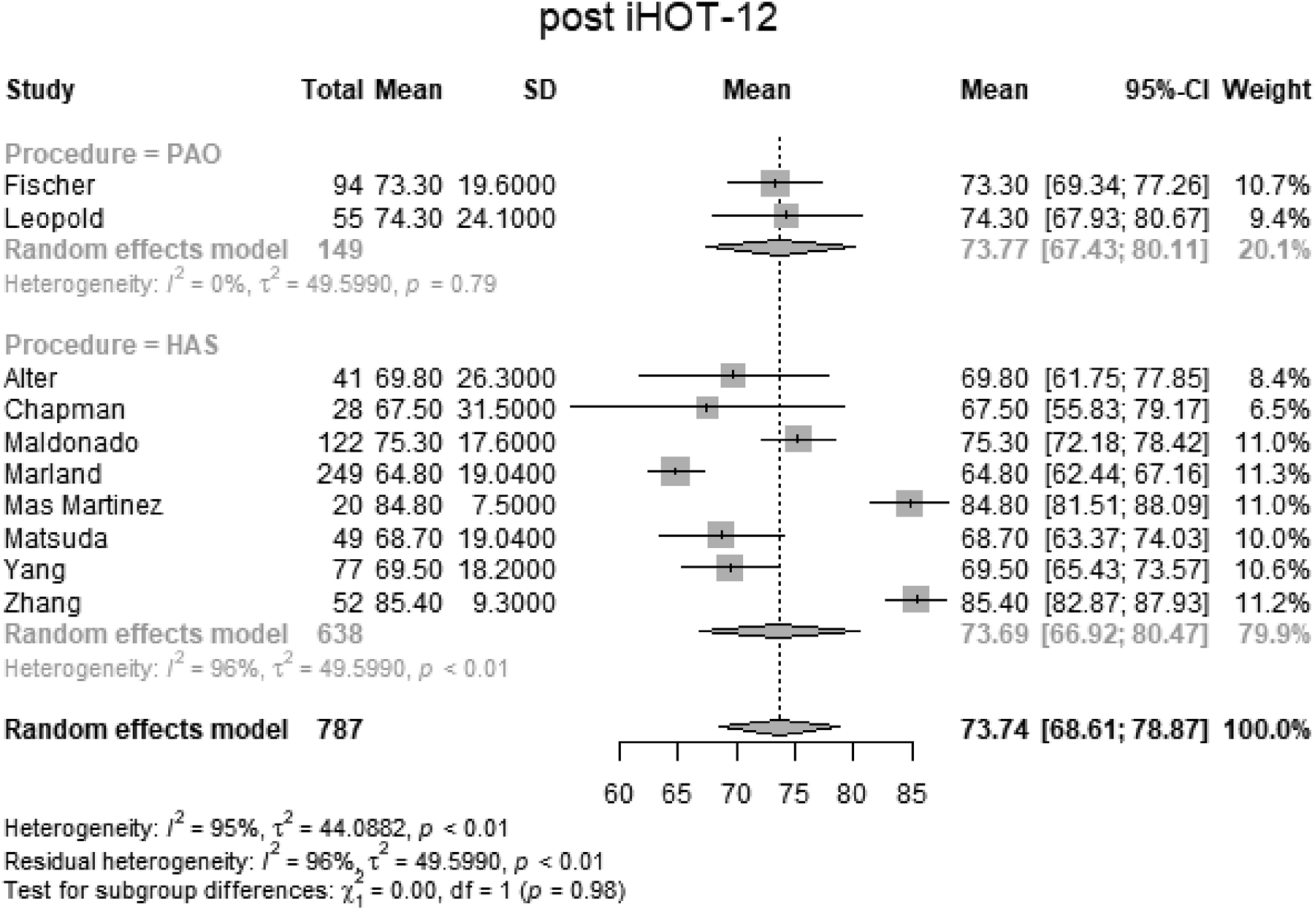
Forest plot of post‐operative iHOT‐12. CI, confidence interval; iHOT, International Hip Outcome Tool; SD, standard deviation.

Data from 787 patients from 10 primary studies were pooled (Figure 10, Table 4), with the PAO group consisting of 149 patients and the HAS group consisting of 638 patients. The mean iHOT‐12 of the PAO group was 73.77 points (mean: 73.77, CIs: 67.43–80.11; *I* ^2^ = 0%; *τ* ^2^ = 49.59, *p* = 0.79). The mean iHOT‐12 of the HAS group was 73.7 points (mean: 73.69, CIs 66.92–80.47; *I* ^2^ = 96%; *τ* ^2^ = 49.59, *p* < 0.01). The test for subgroup differences showed no statistically significant difference between the PAO group and the HAS group in post‐operative iHOT‐12 (*χ* ^2^ = 0.00; df = 1; *p* = 0.98).

#### Change in functional outcome

##### Change in mHHS

**Figure 11:**
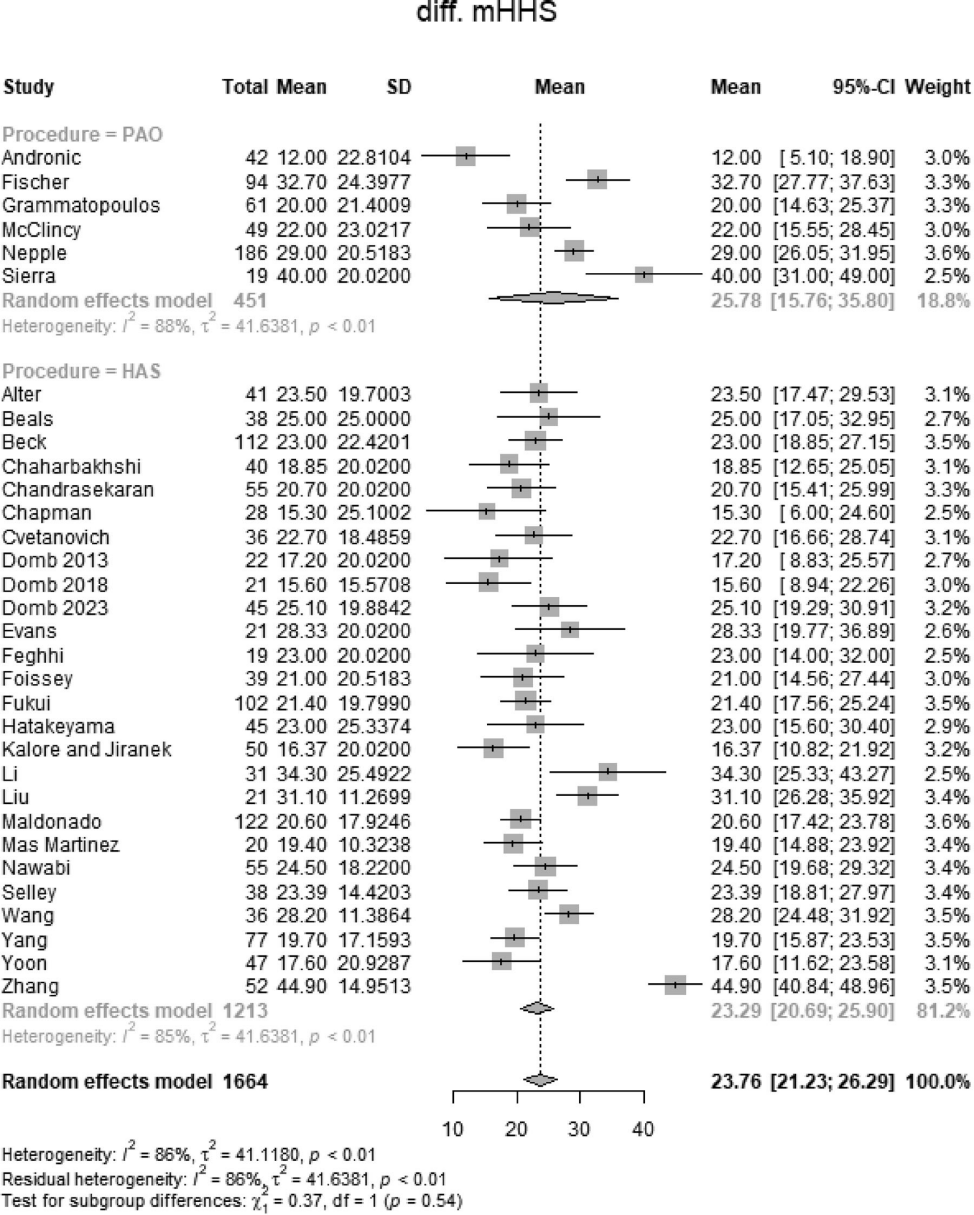
Forest plot of the change in mHHS. CI, confidence interval; mHHS, modified Harris Hip Score; SD, standard deviation*.*

Data from 1664 patients from 32 primary studies were pooled (Figure 11, Table 4), with the PAO group consisting of 451 patients and the HAS group consisting of 1213 patients. The mean change in mHHS of the PAO group was 25.8 points (mean: 25.78, CIs 15.78–35.80; *I* ^2^ = 88%; *τ* ^2^ = 41.64, *p* < 0.01). The mean change in mHHS of the HAS group was 23.3 points (mean: 23.29, CIs: 20.69–25.90; *I* ^2^ = 85%; *τ* ^2^ = 41.64, *p* < 0.01). The test for subgroup differences showed no statistically significant difference between the PAO group and the HAS group in the change in mHHS (*χ* ^2^ = 0.37; df = 1; *p* = 0.54).

##### Change in iHOT‐12

**Figure 12:**
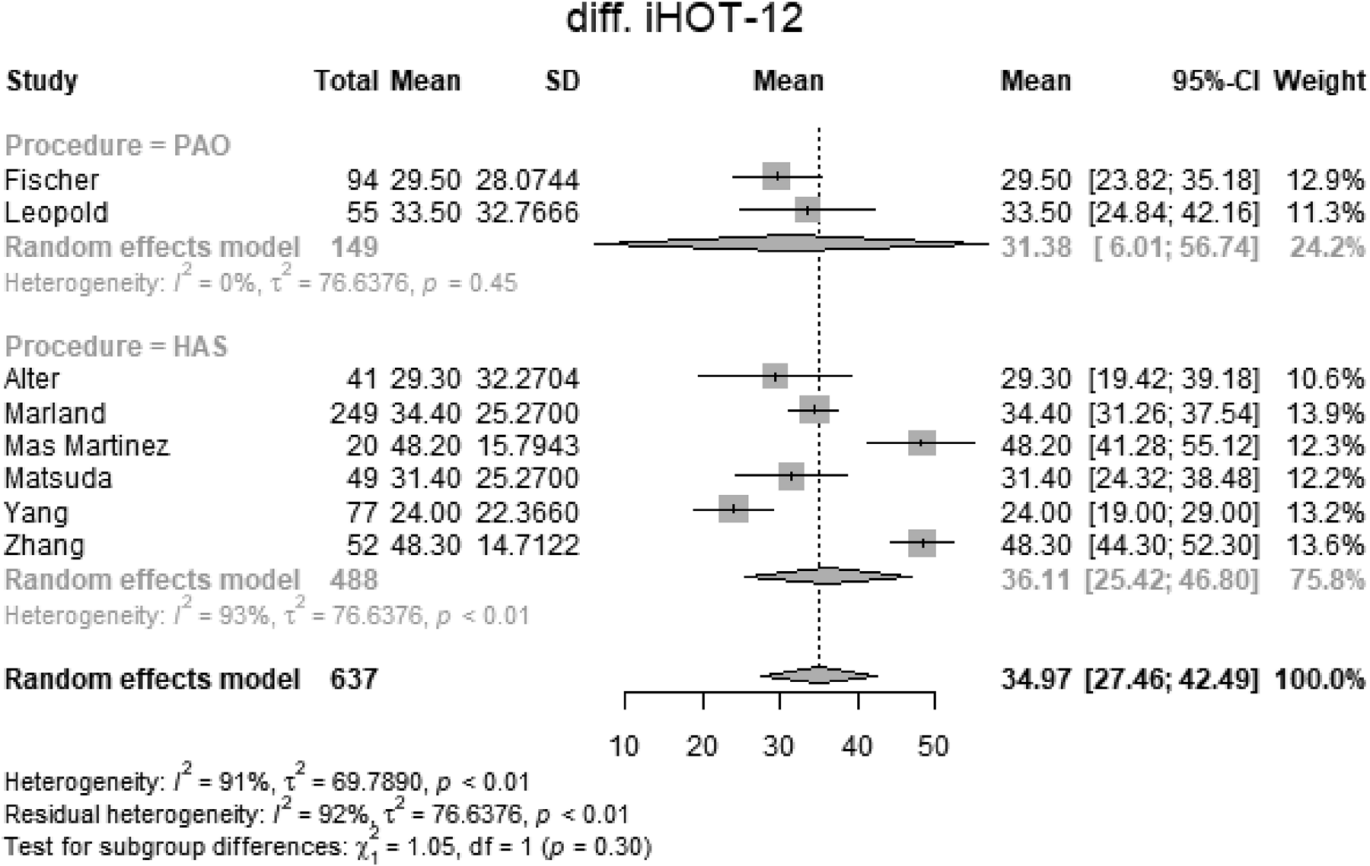
Forest plot of the change in iHOT‐12. CI, confidence interval; iHOT, International Hip Outcome Tool; SD, standard deviation.

Data from 637 patients from eight primary studies were pooled (Figure 12, Table 4), with the PAO group consisting of 149 patients and the HAS group consisting of 488 patients. The mean change in iHOT‐12 of the PAO group was 31.4 points (mean: 31.38, CIs 6.01–56.74; *I* ^2^ = 0%; *τ* ^2^ = 76.64, *p* = 0.45). The mean change in iHOT‐12 of the HAS group was 36.1 points (mean: 36.11, CIs: 25.42–46.8; *I* ^2^ = 93%; *τ* ^2^ = 76.64, *p* < 0.01). The test for subgroup differences showed no statistically significant difference between the PAO group and the HAS group in the change in iHOT‐12 (*χ* ^2^ = 1.05; df = 1; *p* = 0.30).

##### MCID of post‐operative functional outcome scores

**Figure 13:**
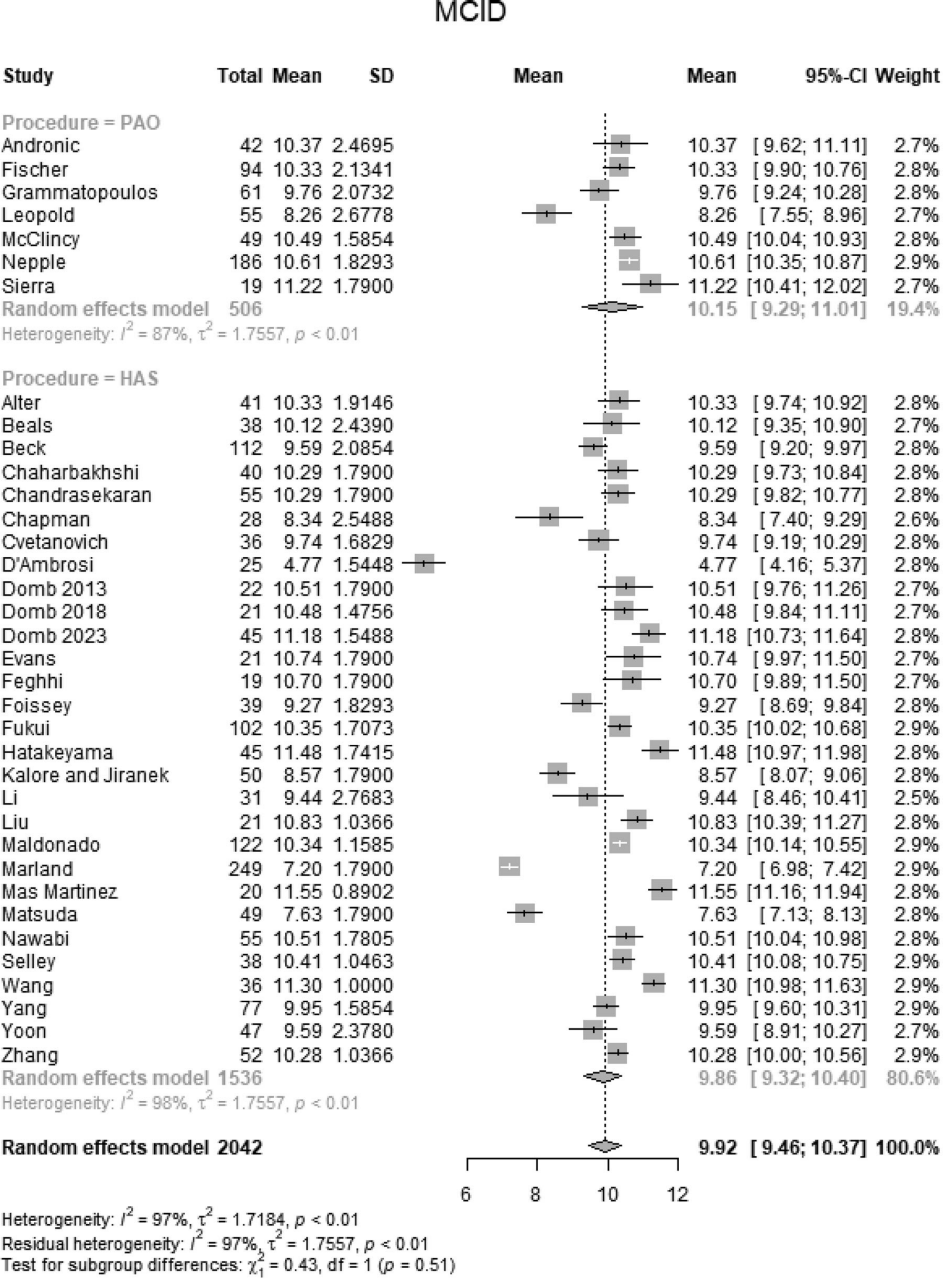
Forest plot of MCID. CI, confidence interval; MCID, minimal clinically important difference; SD, standard deviation*.*

Data from 2042 patients from 36 primary studies were pooled (Figure 13, Table 4), with the PAO group consisting of 506 patients and the HAS group consisting of 1536 patients. The mean MCID of post‐operative functional outcome scores of the PAO group was 10.2 points (mean: 10.15, CIs: 9.29–11.01; *I* ^2^ = 87%; *τ* ^2^ = 1.76, *p* < 0.01). The mean MCID of post‐operative functional outcome scores of the HAS group was 9.9 points (mean: 9.86, CIs: 9.32–10.40; *I* ^2^ = 98%; *τ* ^2^ = 1.76, p < 0.01). The test for subgroup differences showed no statistically significant difference between the PAO group and the HAS group in MCID of post‐operative functional outcome scores (*χ* ^2^ = 0.43; df = 1; *p* = 0.51).

##### Reoperation

**Figure 14:**
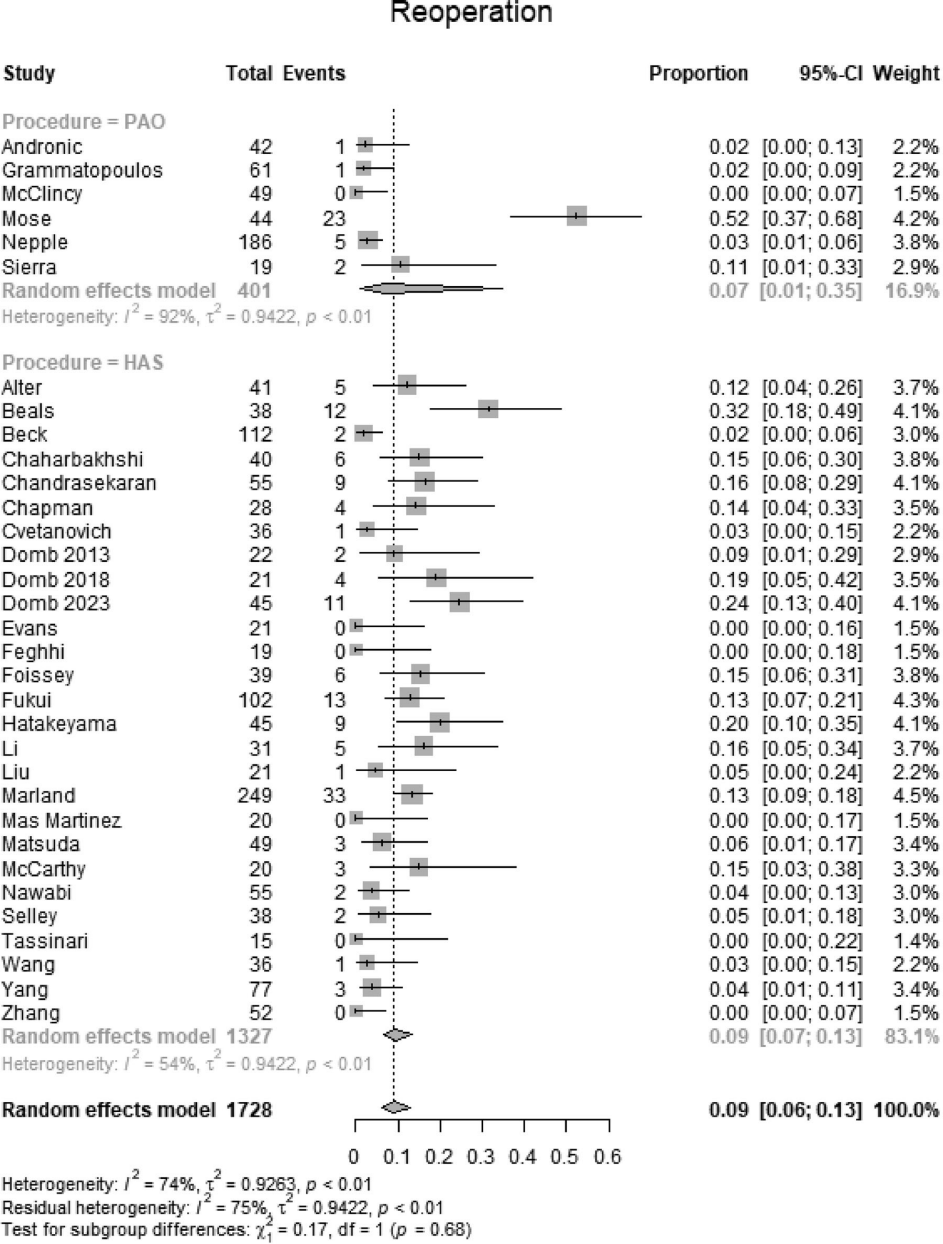
Forest plot of reoperation. CI, confidence interval.

Data from 1728 patients from 33 primary studies were pooled (Figure 14, Table 4), with the PAO group consisting of 401 patients and the HAS group consisting of 1327 patients. In the PAO group, 32 patients had a reoperation (rate: 0.07, CIs: 0.01–0.35; *I* ^2^ = 92%; *τ* ^2^ = 0.42, *p* < 0.01). In the HAS group, 131 patients (rate: 0.09, CIs 0.07–0.13; *I* ^2^ = 54%; *τ* ^2^ = 0.94, *p* < 0.01). The test for subgroup differences showed no statistically significant difference between the PAO group and the HAS group in reoperation (*χ* ^2^= 0.17; df = 1; *p* = 0.68).

##### Complications

**Figure 15:**
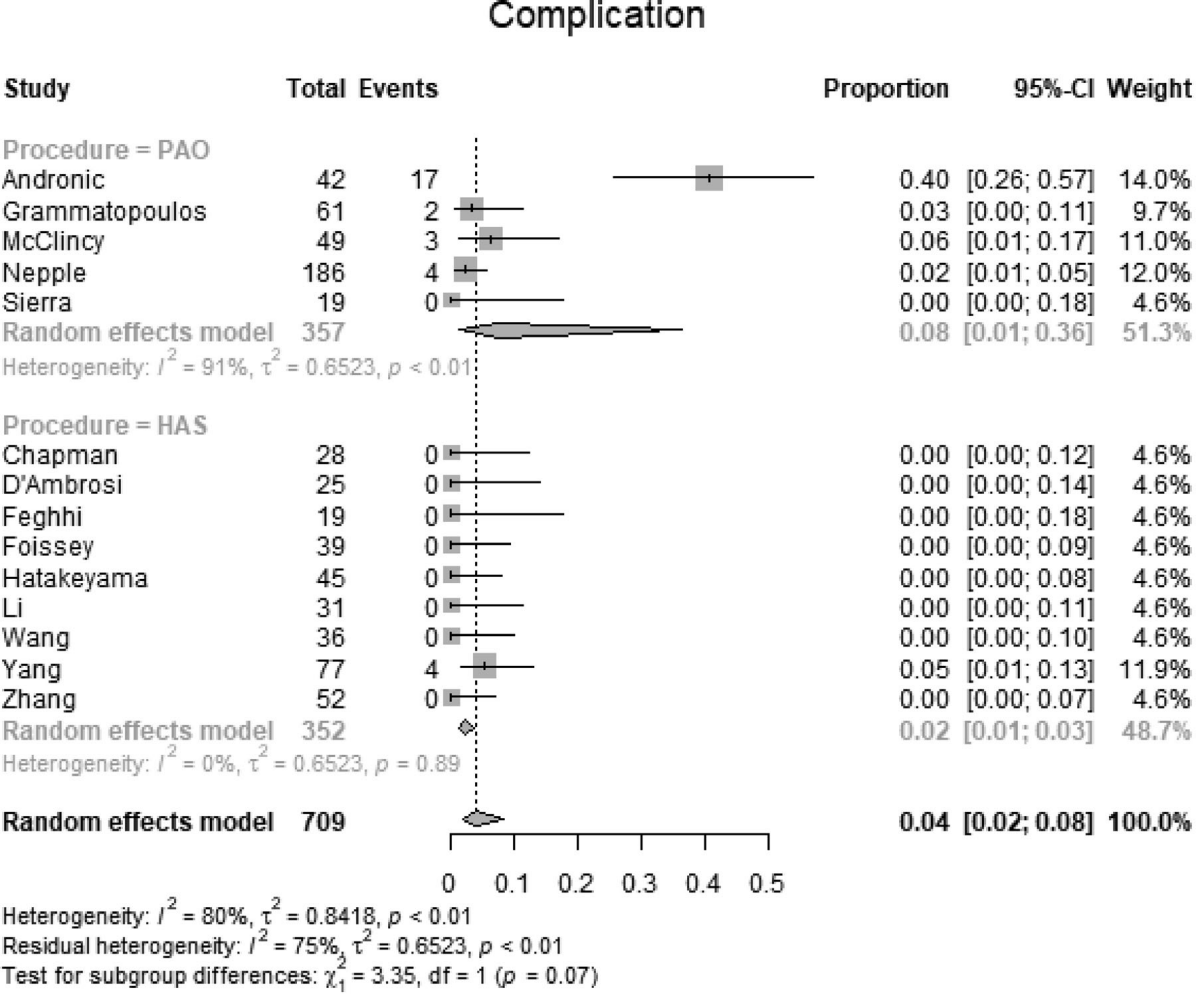
Forest plot of complications. CI, confidence interval.

Data from 709 patients from 14 primary studies were pooled (Figure 15, Table 4), with the PAO group consisting of 357 patients and the HAS group consisting of 352 patients. In the PAO group, 26 patients had a complication (rate: 0.08, CIs: 0.01–0.36; *I* ^2^ = 91%; *τ* ^2^ = 0.65, *p* < 0.01). In the HAS group, four patients (rate: 0.02, CIs 0.01–0.03; *I* ^2^ = 0%; *τ* ^2^ = 0.65, *p* = 0.89). The test for subgroup differences showed no statistically significant difference between the PAO group and the HAS group in complication (*χ* ^2^ = 3.35; df = 1; *p* = 0.07).

##### Additional results

The sensitivity analysis compared studies defining BDDH as 20–25° with the full set of included studies. Significant differences were observed only in post‐operative iHOT‐12, post‐operative HOS‐ADL and the change in iHOT‐12 (Table 5). A set of additional results is available in Figures [Link], [Link], [Link], [Link], [Link], [Link], [Link], [Link], [Link], [Link], [Link], [Link], [Link], [Link], [Link], [Link], [Link], [Link], [Link], [Link], [Link], [Link], [Link], [Link], [Link], [Link], [Link], [Link], [Link], [Link], [Link], [Link], [Link], [Link], [Link], [Link], [Link], [Link], [Link], [Link], [Link], [Link], [Link], [Link]. Preoperative functional and pain scores are presented in Figures [Link], [Link], [Link], [Link], [Link], [Link], [Link], while changes in these scores are shown in Figures [Link], [Link], [Link], [Link], [Link]. Post‐operative functional and pain scores are presented in Figures [Link], [Link], [Link], [Link], [Link]. The distribution of different types of complications is presented in Figures [Link], [Link], [Link], [Link], [Link]. Corresponding funnel plots can be found in Figures [Link], [Link], [Link], [Link], [Link], [Link], [Link], [Link], [Link], [Link], [Link], [Link], [Link], [Link], [Link], [Link], [Link], [Link], [Link], [Link], [Link], [Link].

**Table 5:** Subgroup analysis comparing an LCEA range of 20–25° with broader ranges.

	Primary studies, *N*	Patients, *N*	Mean	CIs	*τ* ^2^	*I* ^2^	Heterogeneity *p*	Egger bias	Egger *p*	Difference *p*
Preoperative										
mHHS	31	1614	60.76	57.89 to 63.62	57.03	0.94	<0.01*	0.03	0.99	0.43
WOMAC	4	199	43.81	12.20 to 75.42	391.75	1.00	<0.01*	3.00	0.94	0.76
NAHS	14	619	59.80	55.49 to 64.10	50.47	0.92	<0.01*	1.31	0.65	0.18
iHOT‐12	8	637	38.90	34.72 to 43.08	19.97	0.92	<0.01*	4.13	0.25	0.25
HOS‐ADL	15	685	67.68	65.07 to –70.29	17.52	0.81	<0.01*	−0.89	0.68	0.68
HOS‐SSS	20	938	44.86	41.65 to 48.06	38.89	0.84	<0.01*	1.03	0.63	0.87
VAS	9	476	6.04	5.48 to 6.60	0.43	0.84	<0.01*	−0.64	0.81	0.19
Post‐operative										
mHHS	31	1614	84.98	83.01 to 86.94	24.62	0.84	<0.01*	−1.10	0.36	0.33
WOMAC	4	199	44.92	−20.84 to 110.67	1704.38	1.00	<0.01*	−10.95	0.88	0.53
NAHS	14	619	82.97	77.61 to 88.33	80.93	0.97	<0.01*	−2.49	0.54	0.81
iHOT‐12	10	787	73.74	68.61 to 78.87	44.09	0.95	<0.01*	−1.56	0.70	0.02**
HOS‐ADL	15	685	89.20	86.69 to 91.70	16.69	0.87	<0.01*	−3.99	0.05**	0.03**
HOS‐SSS	20	938	76.64	73.88 to 79.39	25.50	0.68	<0.01*	0.24	0.82	0.12
VAS	9	476	2.17	1.66 to 2.69	0.37	0.74	0.02*	1.60	0.46	0.92
Change in										
mHHS	31	1614	24.01	21.44 to 26.57	40.84	0.85	<0.01*	−1.40	0.36	0.72
WOMAC	4	199	1.03	−34.79 to 36.85	499.77	1.00	<0.01*	−0.18	0.99	0.34
NAHS	14	619	22.88	18.51 to 27.26	47.91	0.83	<0.01*	0.25	0.93	0.20
iHOT‐12	8	637	34.97	27.46 to –42.49	69.79	0.91	<0.01*	−1.49	0.69	0.02**
HOS‐ADL	15	685	21.22	18.21 to 24.24	22.19	0.72	<0.01*	2.43	0.23	0.25
HOS‐SSS	20	938	31.24	27.13 to 35.36	59.61	0.69	<0.01*	1.30	0.27	0.24
VAS	9	476	−3.84	−4.78 to −2.91	1.25	0.87	<0.01*	0.11	0.97	0.65
MCID										
	35	1992	9.95	9.49 to 10.42	1.72	0.97	<0.01*	0.93	0.73	0.90
Reoperation and complications										
Reoperation	32	1708	0.09	0.06 to 0.13	0.95	0.75	<0.01*	−2.40	0.02*	0.16
Total complications	14	709	0.04	0.02 to 0.08	0.84	0.80	<0.01*	−2.99	0.03***	0.21
Infection	11	529	0.02	0.01 to –0.03	0.04	0.00	0.99	−0.39	0.45	0.23
Nerve injury	11	413	0.04	0.01 to 0.10	1.03	0.78	<0.01*	−3.09	0.03*	0.26
Heterotopic ossifications	11	397	0.02	0.01 to 0.02	0.01	0.00	1.00	−0.55	0.32	0.13

*Note*: The column ‘difference *p*’ indicates statistically significant differences between the groups.

Abbreviations: CI, confidence interval; HOS‐ADL, Hip Outcome Score – Activities of Daily Living; HOS‐SSS, Hip Outcome Score ‐ Sports Subscale; iHOT, International Hip Outcome Tool; LCEA, lateral centre‐edge angle; mHHS, modified Harris Hip Score; NAHS, Non‐Arthritic Hip Score; VAS, visual analogue score; WOMAC, Western Ontario and McMasters Universities Osteoarthritis Index.

* Statistically significant.

** Very statistically significant.

*** Highly statistically significant.

## DISCUSSION

### Main results

To the best of our knowledge, this is the first extensive meta‐analysis comparing the outcomes of PAO and HAS in patients with BDDH. Our findings indicate no statistically significant differences in PROMs between the two procedures, suggesting comparable short‐ to mid‐term clinical effectiveness. However, the absolute mean values for nearly all parameters favour PAO, which may indicate a potential advantage that warrants further investigation. It is crucial to acknowledge the fundamental difference between the two procedures: PAO addresses the underlying structural deficiency and provides a definitive anatomical correction of dysplasia, whereas HAS primarily targets intra‐articular pathology, potentially offering only temporary symptom relief. The sensitivity analysis revealed subtle differences in outcomes when stratifying patients with an acetabular index between 20° and 25°, indicating the necessity for a more standardized definition of BDDH in future research.

### Functional outcomes and pain relief

Post‐operative functional outcomes, as assessed by the modified Harris Hip Score (mHHS) and International Hip Outcome Tool (iHOT‐12), showed substantial improvements in both PAO and HAS groups. The PAO group exhibited a mean mHHS of 85.7, while the HAS group had a mean mHHS of 84.2, with no significant difference between groups (*p* = 0.46). Similarly, iHOT‐12 scores were nearly identical between the two groups (*p* = 0.98). These findings align with previous systematic reviews [3, 27, 38] that reported meaningful PROM improvements following both procedures but lacked direct statistical comparisons. The absence of a clear superiority suggests that both interventions effectively address hip instability and pain associated with BDDH.

### Change in functional scores and clinical significance

The change in mHHS from baseline was comparable between the PAO (25.8 points) and HAS (23.3 points) groups (*p* = 0.54), indicating substantial improvement following both procedures. Similarly, the change in iHOT‐12 showed no significant difference (*p* = 0.30). The MCID analysis, which assesses whether the observed improvements are clinically meaningful, further confirmed the lack of significant differences between groups (*p* = 0.51). These findings suggest that while both PAO and HAS provide meaningful symptom relief, neither procedure is definitively superior in terms of functional gains. It is important to emphasize that the absolute mean values for nearly all parameters favour PAO, potentially indicating an advantage that warrants further investigation.

### Reoperation and complication rates

A key point of debate in BDDH treatment is the durability of PAO and HAS over time. Reoperation rates were comparable between the PAO (7.9%) and HAS (9.9%) groups (*p* = 0.68). The overall complication rates were also similar (*p* = 0.07). However, it is important to note that HAS complications typically involve intra‐articular issues such as labral re‐tears or capsular insufficiency, whereas PAO complications are more likely to involve surgical site infections, nonunion, or loss of reduction. The high heterogeneity (*I* ^2^ > 85% in most comparisons) suggests substantial variability in surgical technique, perioperative management and patient selection, which may influence complication rates.

### Implications for surgical decision‐making

The findings of this meta‐analysis have significant clinical implications. PAO should remain the standard of care for structural correction in patients with more pronounced dysplasia, as it directly addresses acetabular insufficiency. However, HAS has emerged as a viable alternative for select BDDH patients, particularly when intra‐articular pathology is the predominant concern. In HAS, effective capsular management is crucial for optimizing outcomes and preventing iatrogenic instability, highlighting the importance of capsular closure or plication. The high heterogeneity in outcomes emphasizes the need for future studies with standardized surgical protocols and long‐term follow‐up to further refine patient selection criteria and improve treatment decision‐making.

### Interpretation of results

Despite the absence of major differences in clinical outcomes between PAO and HAS in this analysis, PAO remains the only procedure that anatomically corrects dysplasia and restores hip stability. HAS, while providing symptomatic relief in some patients, does not address the underlying biomechanical deficiency and may only serve as a temporizing intervention, particularly in patients with more severe dysplasia. This distinction is critical, as long‐term studies are needed to clarify whether patients undergoing HAS eventually progress to PAO or THA at higher rates.

The sensitivity analysis highlighted that patients with a LCEA of 20–25° exhibited minor outcome differences compared to those outside this range. This finding underscores the need for a uniform and precise definition of BDDH. To improve the consistency of future research, we recommend restricting the definition of BDDH strictly to an LCEA range of 20–25° rather than including broader ranges that might encompass varying pathophysiological subgroups.

### Combined approach

In select cases, a combined approach may be considered, wherein PAO addresses the structural insufficiency and HAS is utilized to treat associated intra‐articular pathology, such as labral tears or femoroacetabular impingement. This sequential or staged strategy could be particularly beneficial in patients with both mechanical instability and symptomatic intra‐articular lesions. In this context, PAO serves as the foundational correction for the underlying biomechanical deficiency, whereas HAS functions as a supportive intervention to manage the secondary consequences arising from this primary pathology. While current literature on combined procedures in BDDH is limited, future studies should investigate whether such multimodal interventions may improve outcomes in anatomically borderline or functionally complex cases.

### Limitations and strengths

One key limitation of this meta‐analysis is the heterogeneity in patient selection criteria across studies, particularly regarding the definition of BDDH. Variability in surgical techniques, follow‐up durations and outcome measures further complicates direct comparisons. Additionally, long‐term data on the progression to osteoarthritis or the need for subsequent surgical interventions remain sparse, preventing definitive conclusions on the durability of HAS versus PAO. Most of the included primary studies had a retrospective design [1, 2, 4, 5, 7, 8, 9, 10, 11, 13, 16, 17, 19, 20, 25, 26, 28, 29, 30, 31, 32, 33, 34, 35, 36, 41, 48, 49, 51, 54, 56, 57, 58], which may introduce selection bias and limit the control over confounding variables. Variability in outcome reporting across studies could affect the consistency and comparability of the pooled results.

Nevertheless, this study offers several strengths. It provides a comprehensive synthesis of available data, offering valuable insights into treatment decision‐making for BDDH. The sensitivity analysis further refines our understanding of the LCEA threshold for defining BDDH, reinforcing the need for a standardized classification. Lastly, by emphasizing the importance of structural correction through PAO compared to symptomatic relief with HAS, our findings guide clinicians in tailoring treatment approaches based on individual patient characteristics and long‐term joint preservation goals.

## CONCLUSIONS

In summary, PAO and HAS yield similar short‐ to mid‐term clinical outcomes in patients with BDDH. The role of HAS in this patient population requires further long‐term evaluation to determine whether it serves as a permanent solution or merely delays the need for structural correction. Importantly, future research should adopt a stricter definition of BDDH (LCEA 20–25°) to improve comparability across studies and improve treatment standardization.

## AUTHOR CONTRIBUTIONS

**Nikolai Ramadanov:** Conceptualization; data curation; formal analysis; investigation; methodology; project administration; resources; software; supervision; validation; visualization; writing—original draft; writing—review and editing. **Maximilian Voss:** Data curation; formal analysis; investigation. **Robert Hable:** Conceptualization; methodology; project administration; resources; software; supervision; validation; visualization; writing—review and editing. **Robert Prill:** Writing—review and editing. **Dobromir Dimitrov:** Writing—review and editing. **Roland Becker:** Writing—review and editing. **Ingo J. Banke:** Writing—review and editing. **Marco Haertlé:** Writing—review and editing. **Sufian S. Ahmad:** Writing—review and editing.

## CONFLICT OF INTEREST STATEMENT

The authors declare no conflicts of interest.

## ETHICS STATEMENT

The ethics statement is not available.

## Supporting information

Figure S1. Forest plot preoperative mHHS. mHHS: modified Harris Hip Score; SD: standard deviation; CI: confidence interval.

Figure S2. Forest plot preoperative WOMAC. WOMAC: Western Ontario and McMaster Universities Osteoarthritis Index; SD: standard deviation; CI: confidence interval.

Figure S3. Forest plot preoperative NAHS. NAHS: Non‐Arthritic Hip Score; SD: standard deviation; CI: confidence interval.

Figure S4. Forest plot preoperative iHOT‐12. iHOT: International Hip Outcome Tool; SD: standard deviation; CI: confidence interval.

Figure S5. Forest plot preoperative HOS‐ADL. HOS‐ADL: Hip Outcome Score ‐ Activities of Daily Living; SD: standard deviation; CI: confidence interval.

Figure S6. Forest plot preoperative HOS‐SSS. HOS‐SSS: Hip Outcome Score – Sport Subscale; SD: standard deviation; CI: confidence interval.

Figure S7. Forest plot preoperative VAS. VAS: Visual Analog Scale; SD: standard deviation; CI: confidence interval.

Figure S8. Forest plot change in WOMAC. WOMAC: Western Ontario and McMaster Universities Osteoarthritis Index; SD: standard deviation; CI: confidence interval.

Figure S9. Forest plot change in NAHS. NAHS: Non‐Arthritic Hip Score; SD: standard deviation; CI: confidence interval.

Figure S10. Forest plot change in HOS‐ADL. HOS‐ADL: Hip Outcome Score ‐ Activities of Daily Living; SD: standard deviation; CI: confidence interval.

Figure S11. Forest plot preoperative HOS‐SSS. HOS‐SSS Hip Outcome Score – Sport Subscale; SD: standard deviation; CI: confidence interval.

Figure S12. Forest plot change in VAS. VAS: Visual Analog Scale; SD: standard deviation; CI: confidence interval.

Figure S13. Forest plot post‐operative WOMAC. WOMAC: Western Ontario and McMaster Universities Osteoarthritis Index; SD: standard deviation; CI: confidence interval.

Figure S14. Forest plot post‐operative NAHS. NAHS: Non‐Arthritic Hip Score; SD: standard deviation; CI: confidence interval.

Figure S15. Forest plot preoperative HOS‐ADL. HOS‐ADL: Hip Outcome Score ‐ Activities of Daily Living; SD: standard deviation; CI: confidence interval.

Figure S16. Forest plot preoperative HOS‐SSS. HOS‐SSS Hip Outcome Score – Sport Subscale; SD: standard deviation; CI: confidence interval.

Figure S17. Forest plot preoperative VAS. VAS: Visual Analog Scale; SD: standard deviation; CI: confidence interval.

Figure S18. Forest plot Infection. CI: confidence interval.

Figure S19. Forest plot Nerve injury. CI: confidence interval.

Figure S20. Forest plot DVT/PE. DVT: deep vein thrombosis; PE: pulmonary embolism; CI: confidence interval.

Figure S21. Forest plot Loss reduction/Nonunion. CI: confidence interval.

Figure S22. Forest plot Heterotopic ossification. CI: confidence interval.

Figure S23. Funnel plot preoperative mHHS. mHHS: modified Harris Hip SD: standard deviation; CI: confidence interval.

Figure S24. Funnel plot preoperative WOMAC. WOMAC: Western Ontario and McMaster Universities Osteoarthritis Index.

Figure S25. Funnel plot preoperative NAHS. NAHS: Non‐Arthritic Hip Score.

Figure S26. Funnel plot preoperative iHOT‐12. iHOT: International Hip Outcome Tool.

Figure S27. Funnel plot preoperative HOS‐ADL. HOS‐ADL: Hip Outcome Score ‐ Activities of Daily Living.

Figure S28. Funnel plot preoperative HOS‐SSS. HOS‐SSS: Hip Outcome Score – Sport Subscale.

Figure S29. Funnel plot preoperative VAS: VAS: Visual Analog Scale.

Figure S30. Funnel plot change in WOMAC. WOMAC: Western Ontario and McMaster Universities Osteoarthritis Index.

Figure S31. Funnel plot change in NAHS. NAHS: Non‐Arthritic Hip Score.

Figure S32. Funnel plot change in HOS‐ADL. HOS‐ADL: Hip Outcome Score ‐ Activities of Daily Living.

Figure S33. Funnel plot change in HOS‐SSS. HOS‐SSS: Hip Outcome Score – Sport Subscale.

Figure S34. Funnel plot change in VAS. VAS: Visual Analog Scale.

Figure S35. Funnel plot post‐operative WOMAC. WOMAC: Western Ontario and McMaster Universities Osteoarthritis Index.

Figure S36. Funnel plot post‐operative NAHS. NAHS: Non‐Arthritic Hip Score.

Figure S37. Funnel plot post‐operative HOS‐ADL. HOS‐ADL: Hip Outcome Score ‐ Activities of Daily Living.

Figure S38. Funnel plot post‐operative HOS‐SSS. HOS‐SSS: Hip Outcome Score – Sport Subscale.

Figure S39. Funnel plot change in VAS. VAS: Visual Analog Scale.

Figure S40. Funnel plot Infection.

Figure S41. Funnel plot Nerve injury.

Figure S42. Funnel plot DVT/PE. DVT: deep vein thrombosis; PE: pulmonary embolism.

Figure S43. Funnel plot Loss reduction/Nonunion.

Figure S44. Funnel plot Heterotopic ossification.

## Data Availability

The data extraction set will be available in the Supporting Information of the published version of this article.
