# Supplementary figures and images for: Periacetabular osteotomy versus hip arthroscopy in patients with borderline developmental dysplasia of the hip: A systematic review and multi‐level meta‐analysis
Source: J Exp Orthop. 2025 Jul 2;12(3):e70311. doi: 10.1002/jeo2.70311 (PMC12221243; doi:10.1002/jeo2.70311)

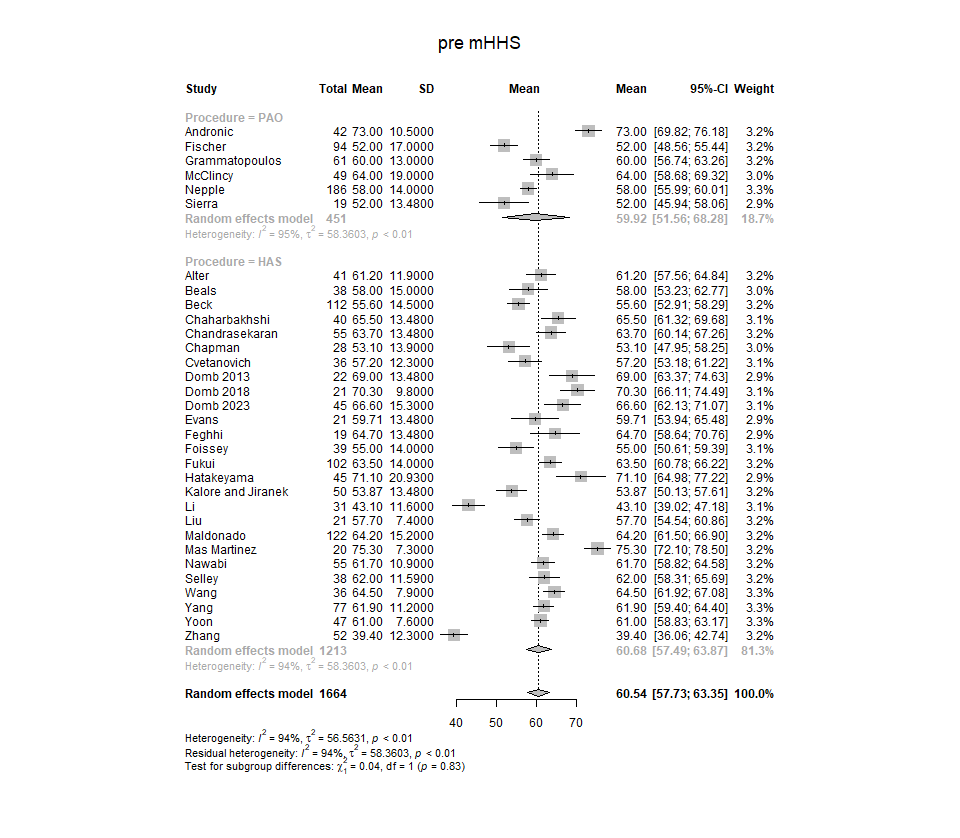

Supplement: Supplementary file 1 — Figure S1. Forest plot preoperative mHHS. mHHS: modified Harris Hip Score; SD: standard deviation; CI: confidence interval. [file JEO2-12-e70311-s029.png]

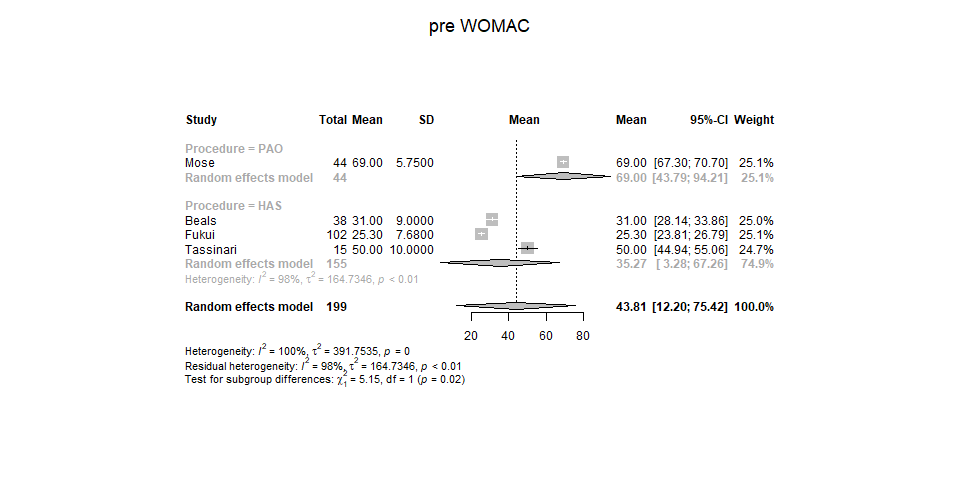

Supplement: Supplementary file 2 — Figure S2. Forest plot preoperative WOMAC. WOMAC: Western Ontario and McMaster Universities Osteoarthritis Index; SD: standard deviation; CI: confidence interval. [file JEO2-12-e70311-s032.png]

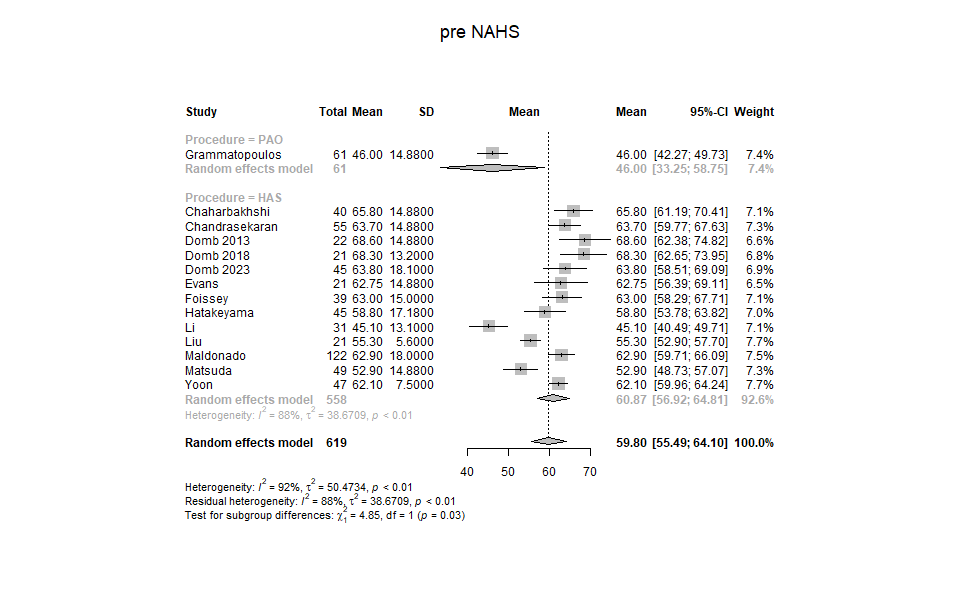

Supplement: Supplementary file 3 — Figure S3. Forest plot preoperative NAHS. NAHS: Non‐Arthritic Hip Score; SD: standard deviation; CI: confidence interval. [file JEO2-12-e70311-s035.png]

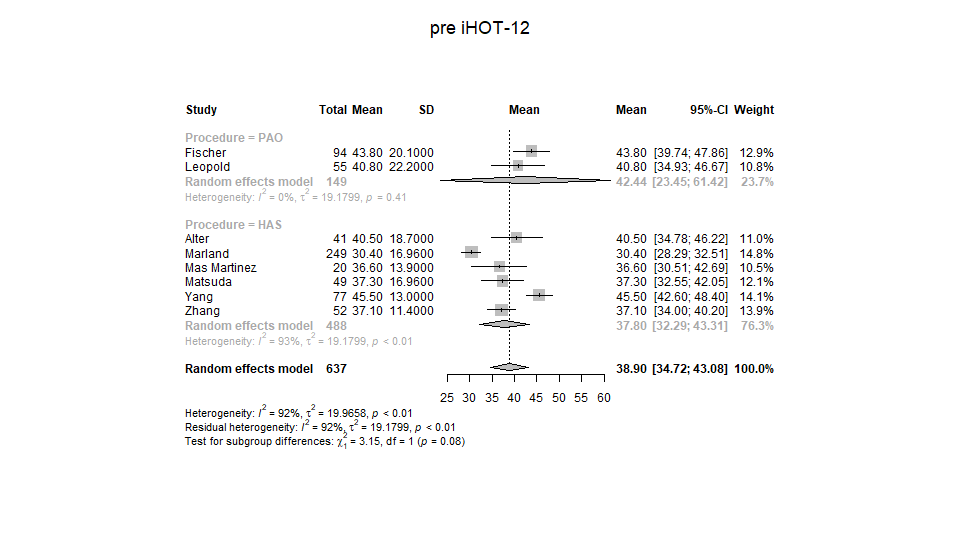

Supplement: Supplementary file 4 — Figure S4. Forest plot preoperative iHOT‐12. iHOT: International Hip Outcome Tool; SD: standard deviation; CI: confidence interval. [file JEO2-12-e70311-s004.png]

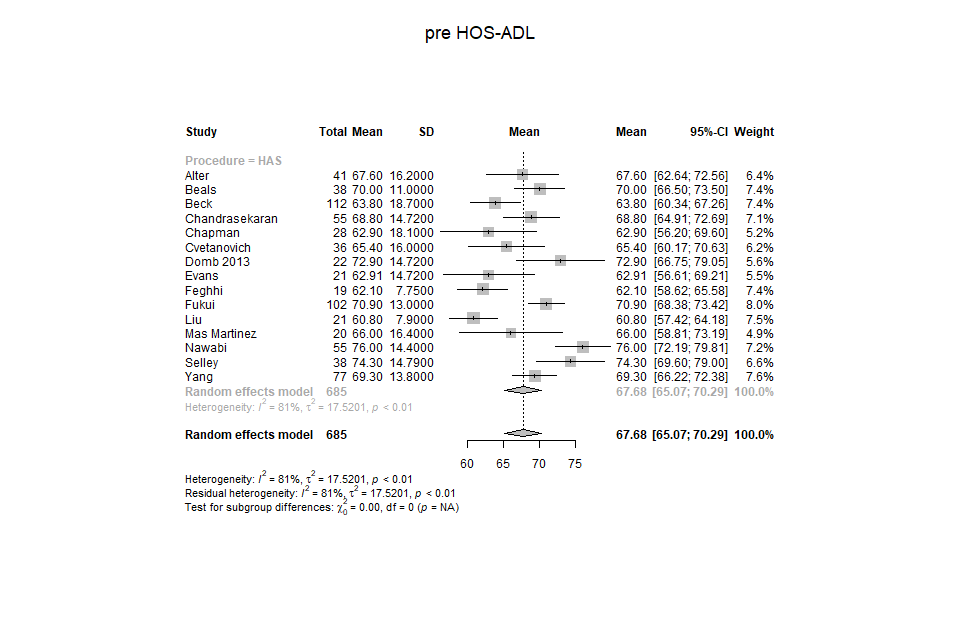

Supplement: Supplementary file 5 — Figure S5. Forest plot preoperative HOS‐ADL. HOS‐ADL: Hip Outcome Score ‐ Activities of Daily Living; SD: standard deviation; CI: confidence interval. [file JEO2-12-e70311-s013.png]

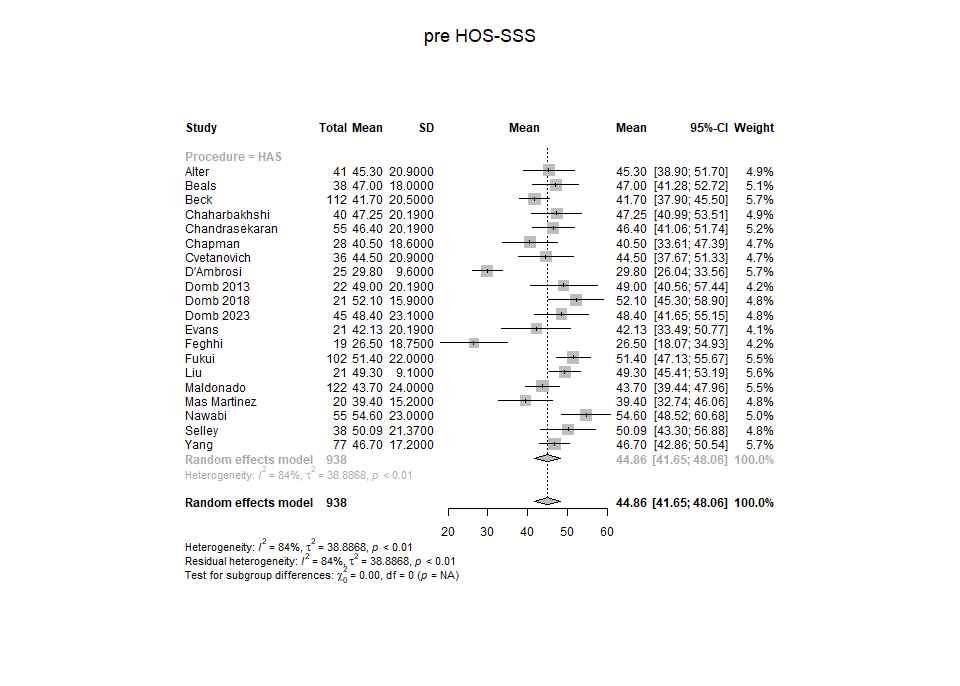

Supplement: Supplementary file 6 — Figure S6. Forest plot preoperative HOS‐SSS. HOS‐SSS: Hip Outcome Score – Sport Subscale; SD: standard deviation; CI: confidence interval. [file JEO2-12-e70311-s008.png]

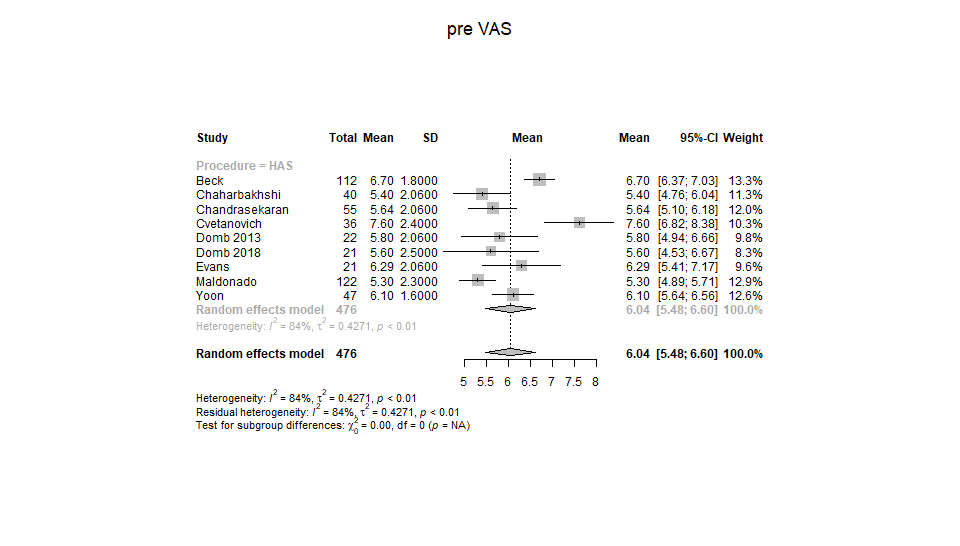

Supplement: Supplementary file 7 — Figure S7. Forest plot preoperative VAS. VAS: Visual Analog Scale; SD: standard deviation; CI: confidence interval. [file JEO2-12-e70311-s017.png]

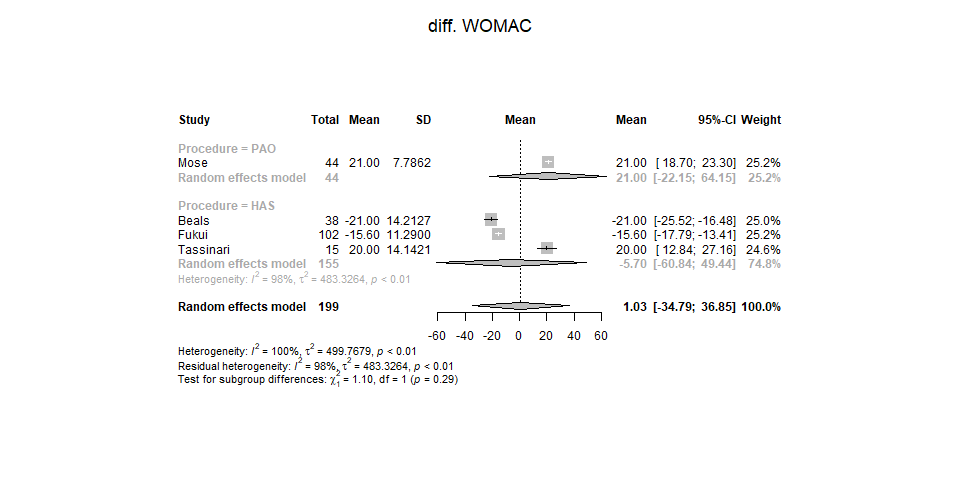

Supplement: Supplementary file 8 — Figure S8. Forest plot change in WOMAC. WOMAC: Western Ontario and McMaster Universities Osteoarthritis Index; SD: standard deviation; CI: confidence interval. [file JEO2-12-e70311-s026.png]

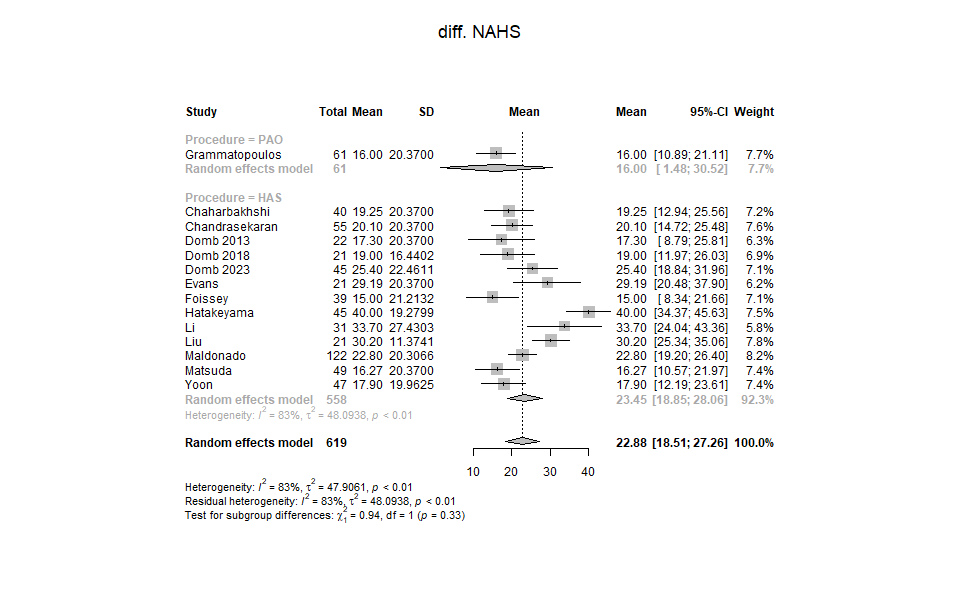

Supplement: Supplementary file 9 — Figure S9. Forest plot change in NAHS. NAHS: Non‐Arthritic Hip Score; SD: standard deviation; CI: confidence interval. [file JEO2-12-e70311-s028.png]

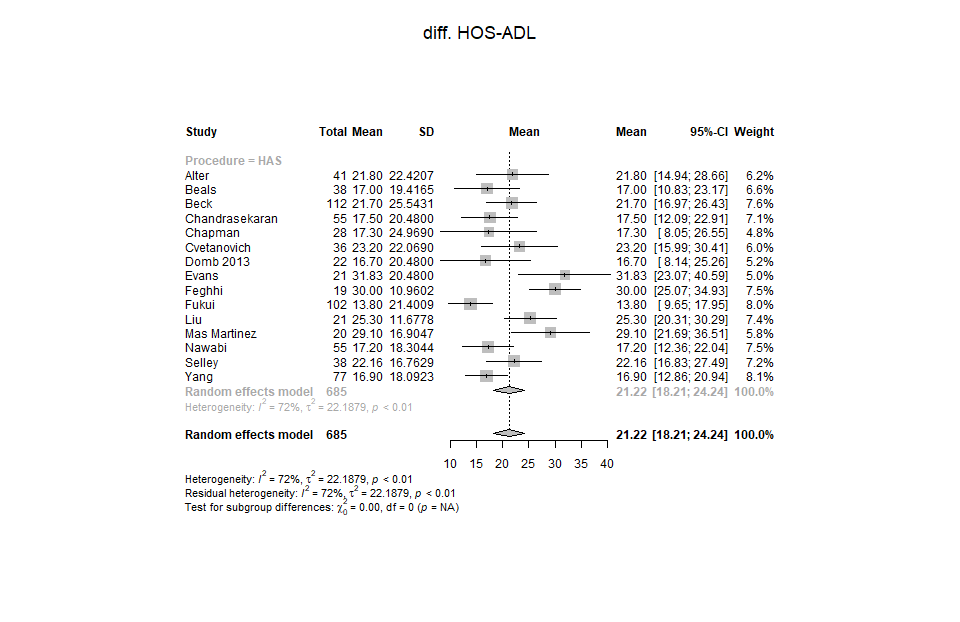

Supplement: Supplementary file 10 — Figure S10. Forest plot change in HOS‐ADL. HOS‐ADL: Hip Outcome Score ‐ Activities of Daily Living; SD: standard deviation; CI: confidence interval. [file JEO2-12-e70311-s012.png]

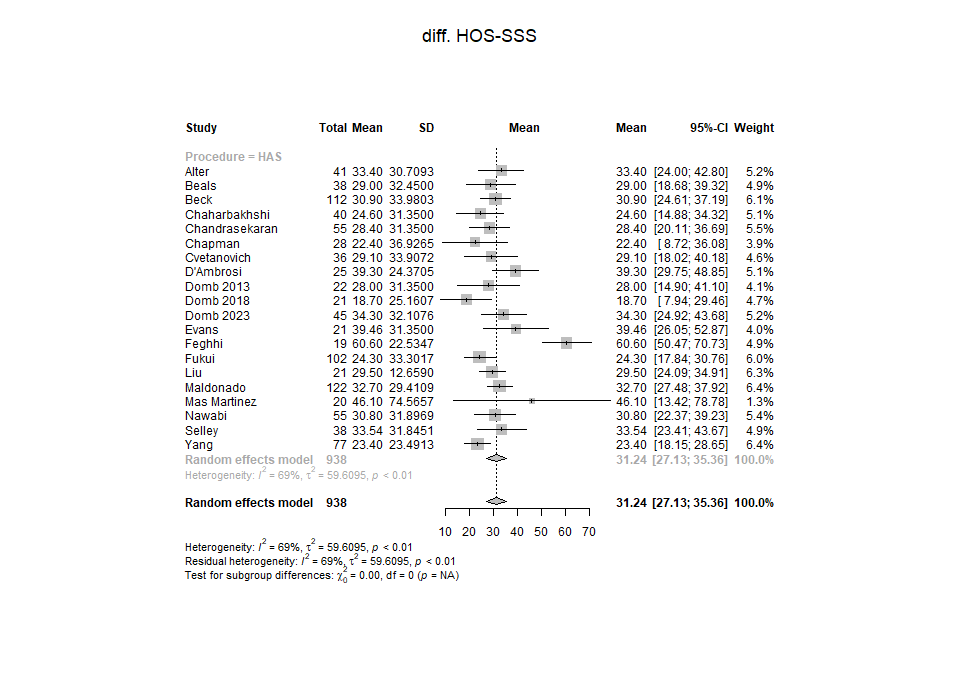

Supplement: Supplementary file 11 — Figure S11. Forest plot preoperative HOS‐SSS. HOS‐SSS Hip Outcome Score – Sport Subscale; SD: standard deviation; CI: confidence interval. [file JEO2-12-e70311-s020.png]

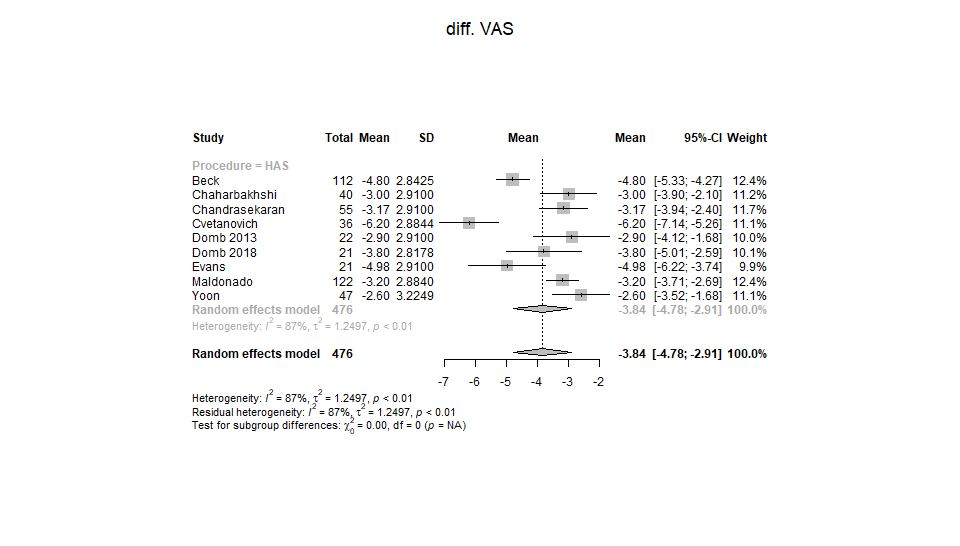

Supplement: Supplementary file 12 — Figure S12. Forest plot change in VAS. VAS: Visual Analog Scale; SD: standard deviation; CI: confidence interval. [file JEO2-12-e70311-s014.png]

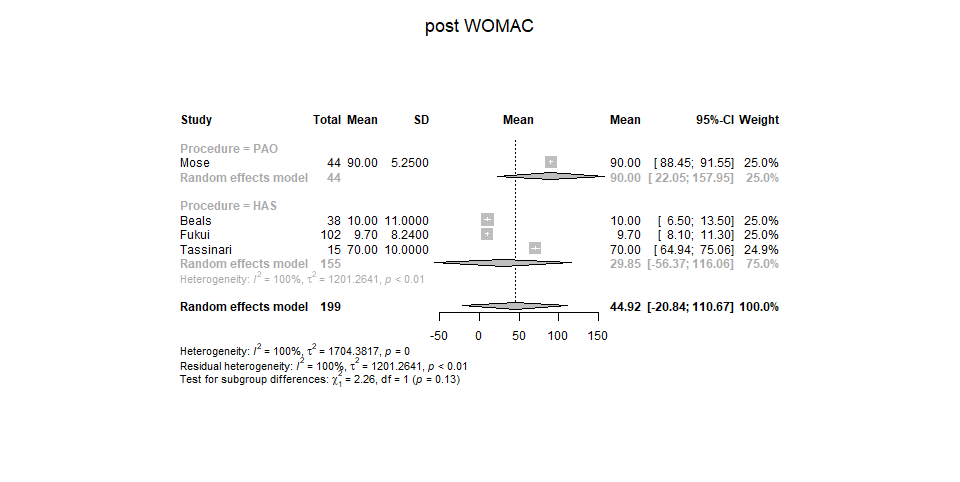

Supplement: Supplementary file 13 — Figure S13. Forest plot post‐operative WOMAC. WOMAC: Western Ontario and McMaster Universities Osteoarthritis Index; SD: standard deviation; CI: confidence interval. [file JEO2-12-e70311-s041.png]

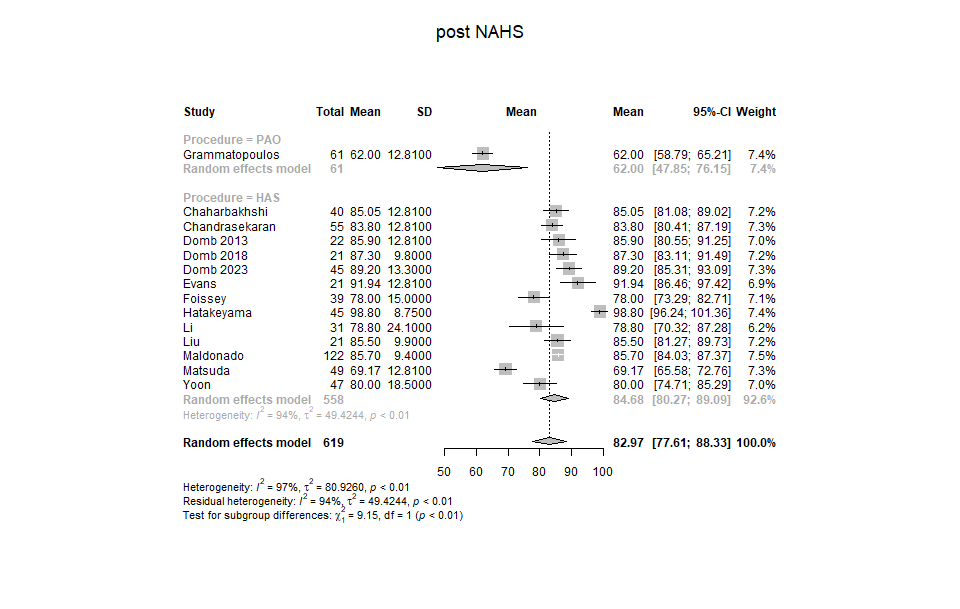

Supplement: Supplementary file 14 — Figure S14. Forest plot post‐operative NAHS. NAHS: Non‐Arthritic Hip Score; SD: standard deviation; CI: confidence interval. [file JEO2-12-e70311-s022.png]

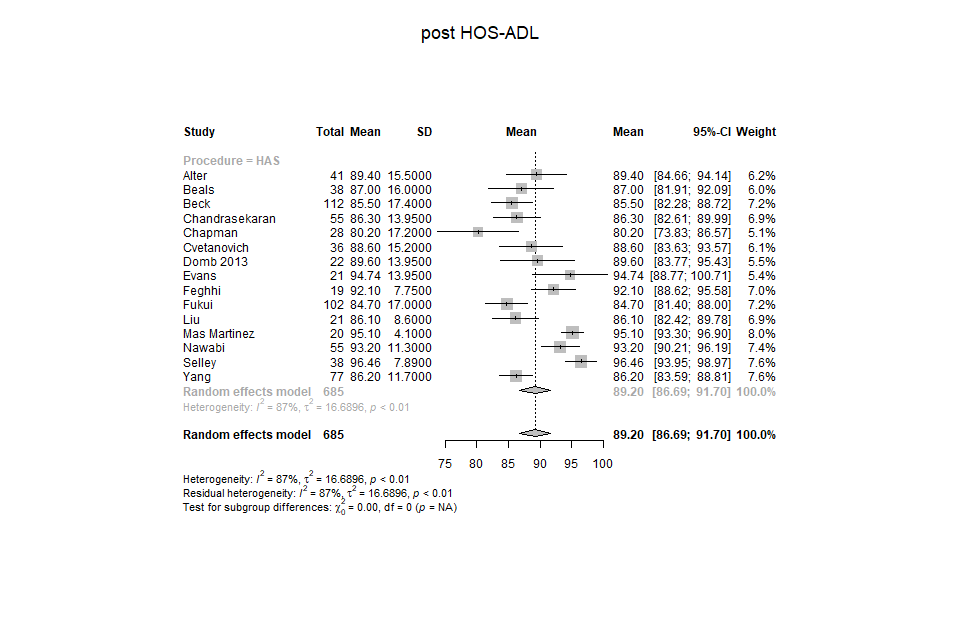

Supplement: Supplementary file 15 — Figure S15. Forest plot preoperative HOS‐ADL. HOS‐ADL: Hip Outcome Score ‐ Activities of Daily Living; SD: standard deviation; CI: confidence interval. [file JEO2-12-e70311-s027.png]

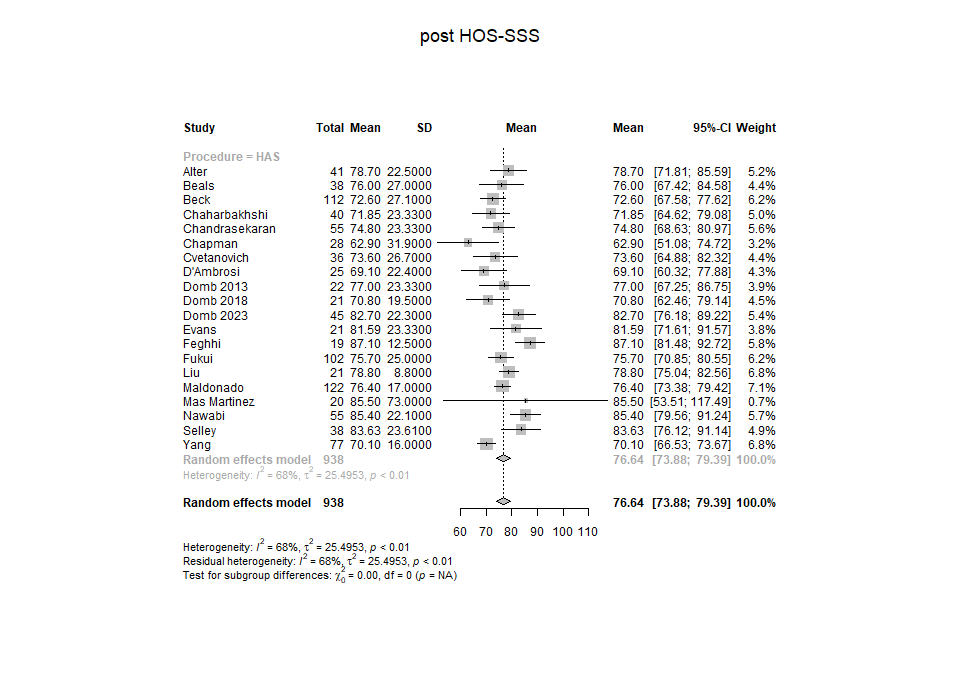

Supplement: Supplementary file 16 — Figure S16. Forest plot preoperative HOS‐SSS. HOS‐SSS Hip Outcome Score – Sport Subscale; SD: standard deviation; CI: confidence interval. [file JEO2-12-e70311-s037.png]

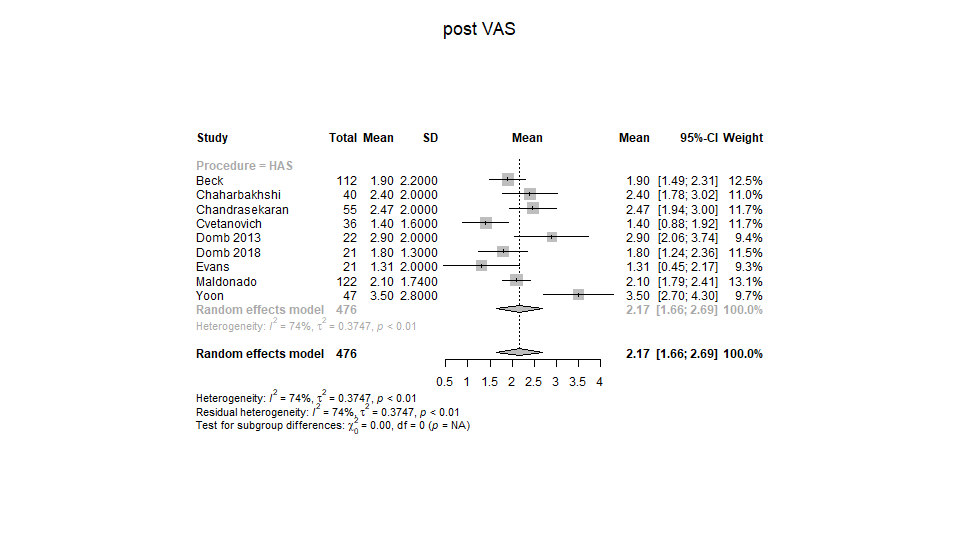

Supplement: Supplementary file 17 — Figure S17. Forest plot preoperative VAS. VAS: Visual Analog Scale; SD: standard deviation; CI: confidence interval. [file JEO2-12-e70311-s040.png]

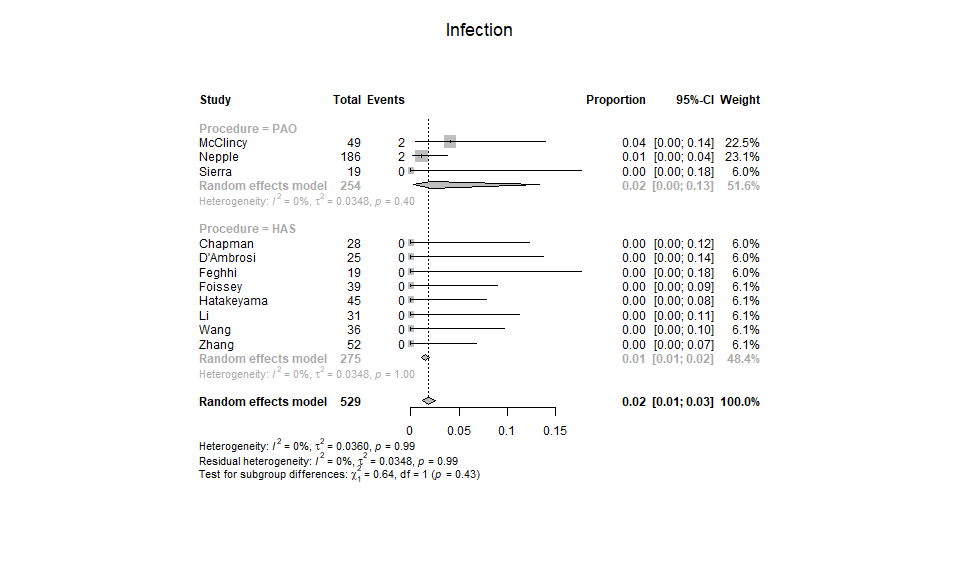

Supplement: Supplementary file 18 — Figure S18. Forest plot Infection. CI: confidence interval. [file JEO2-12-e70311-s043.png]

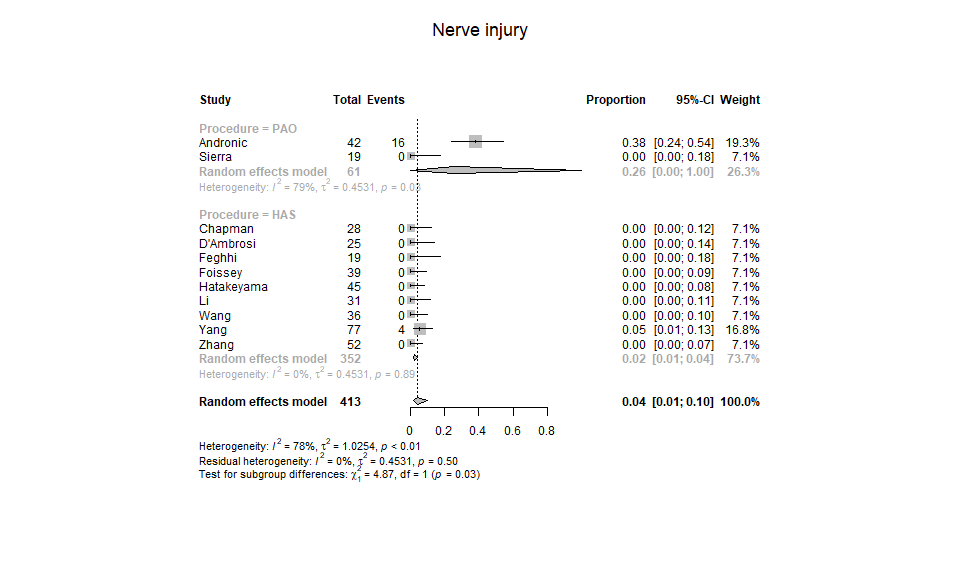

Supplement: Supplementary file 19 — Figure S19. Forest plot Nerve injury. CI: confidence interval. [file JEO2-12-e70311-s009.png]

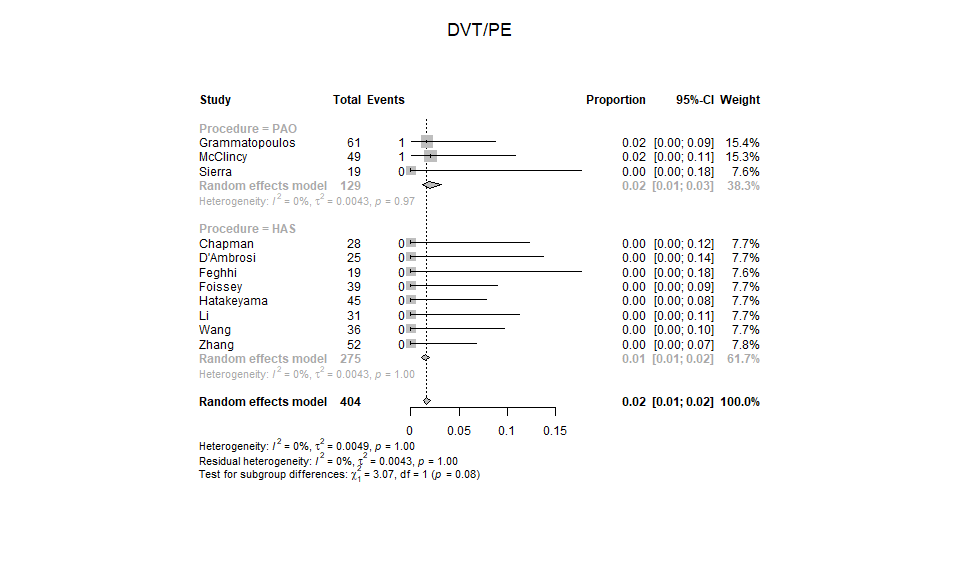

Supplement: Supplementary file 20 — Figure S20. Forest plot DVT/PE. DVT: deep vein thrombosis; PE: pulmonary embolism; CI: confidence interval. [file JEO2-12-e70311-s019.png]

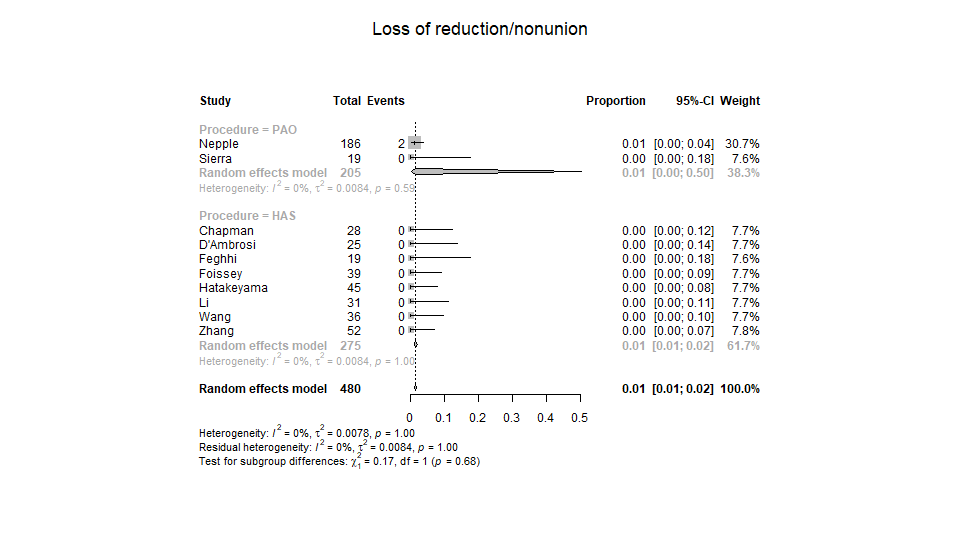

Supplement: Supplementary file 21 — Figure S21. Forest plot Loss reduction/Nonunion. CI: confidence interval. [file JEO2-12-e70311-s030.png]

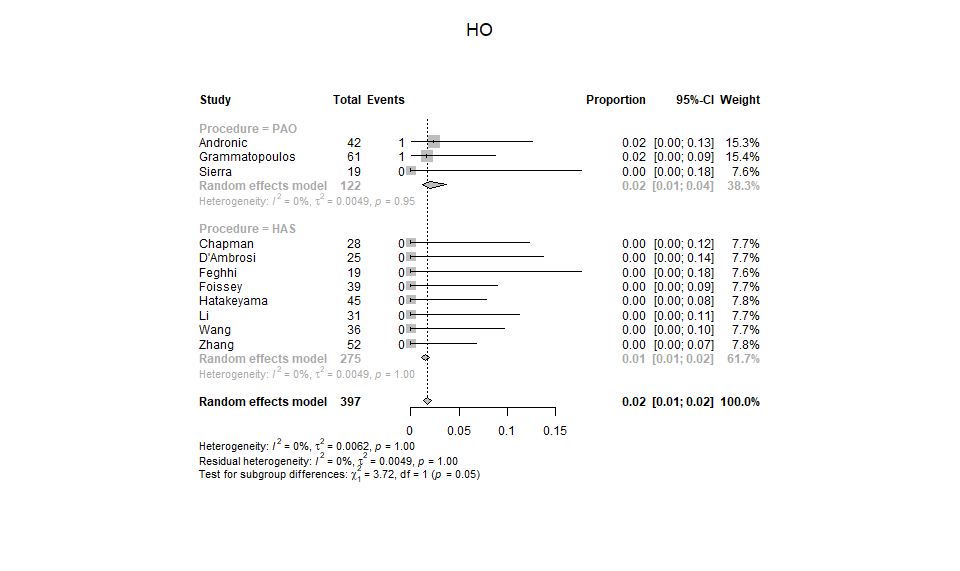

Supplement: Supplementary file 22 — Figure S22. Forest plot Heterotopic ossification. CI: confidence interval. [file JEO2-12-e70311-s036.png]

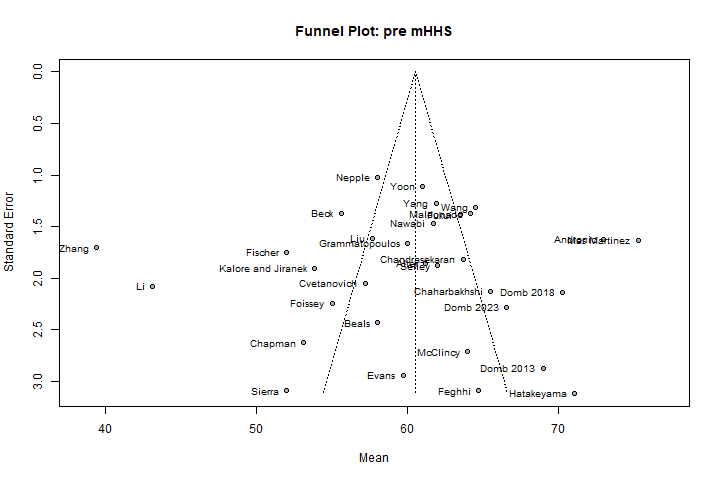

Supplement: Supplementary file 23 — Figure S23. Funnel plot preoperative mHHS. mHHS: modified Harris Hip SD: standard deviation; CI: confidence interval. [file JEO2-12-e70311-s033.png]

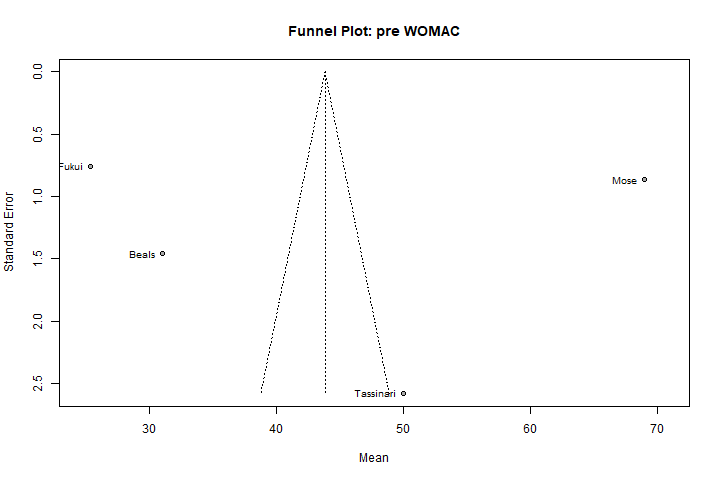

Supplement: Supplementary file 24 — Figure S24. Funnel plot preoperative WOMAC. WOMAC: Western Ontario and McMaster Universities Osteoarthritis Index. [file JEO2-12-e70311-s044.png]

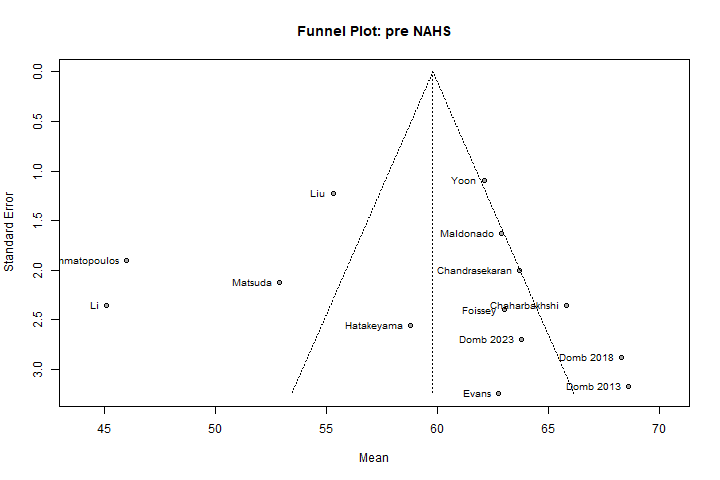

Supplement: Supplementary file 25 — Figure S25. Funnel plot preoperative NAHS. NAHS: Non‐Arthritic Hip Score. [file JEO2-12-e70311-s015.png]

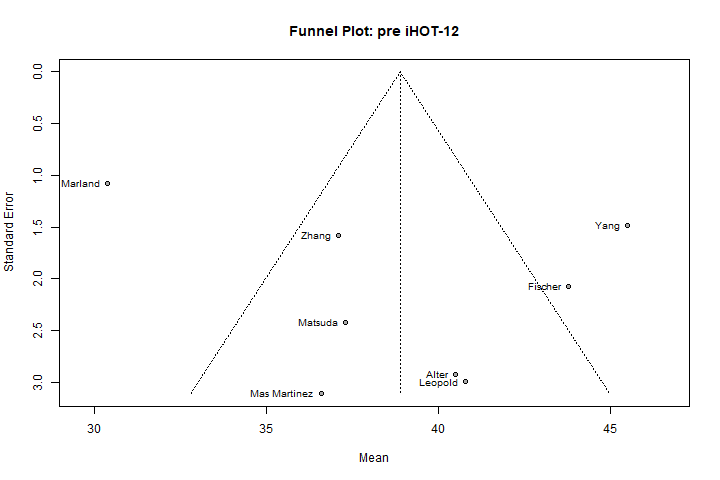

Supplement: Supplementary file 26 — Figure S26. Funnel plot preoperative iHOT‐12. iHOT: International Hip Outcome Tool. [file JEO2-12-e70311-s010.png]

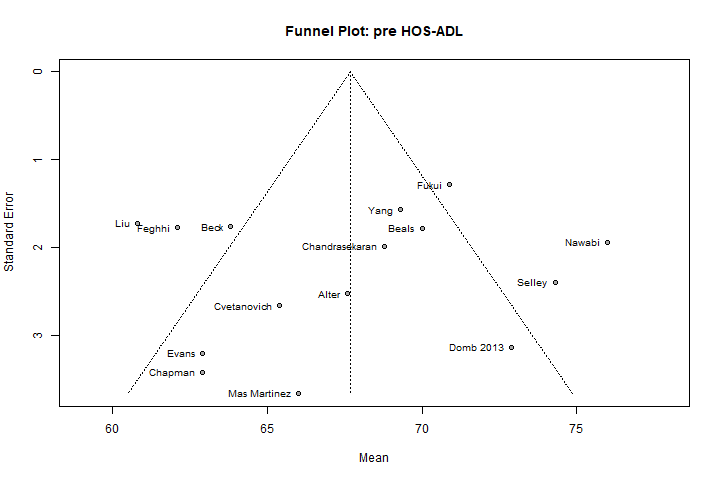

Supplement: Supplementary file 27 — Figure S27. Funnel plot preoperative HOS‐ADL. HOS‐ADL: Hip Outcome Score ‐ Activities of Daily Living. [file JEO2-12-e70311-s005.png]

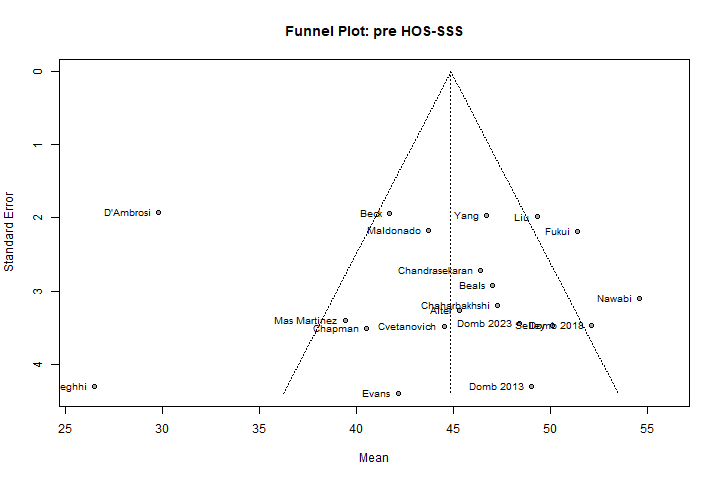

Supplement: Supplementary file 28 — Figure S28. Funnel plot preoperative HOS‐SSS. HOS‐SSS: Hip Outcome Score – Sport Subscale. [file JEO2-12-e70311-s007.png]

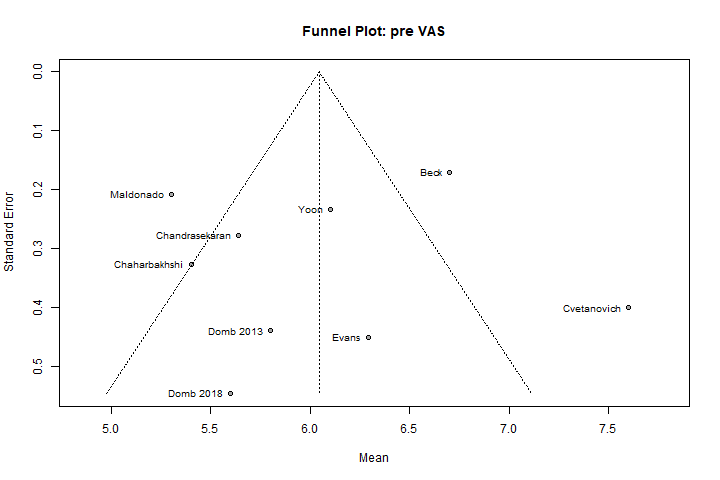

Supplement: Supplementary file 29 — Figure S29. Funnel plot preoperative VAS: VAS: Visual Analog Scale. [file JEO2-12-e70311-s025.png]

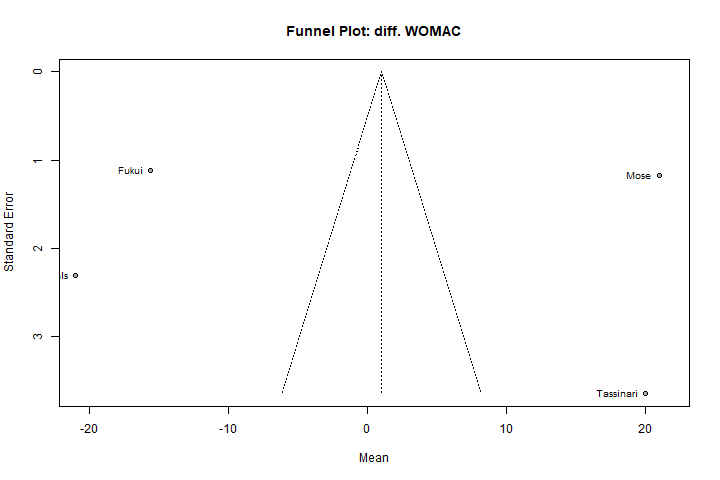

Supplement: Supplementary file 30 — Figure S30. Funnel plot change in WOMAC. WOMAC: Western Ontario and McMaster Universities Osteoarthritis Index. [file JEO2-12-e70311-s002.png]

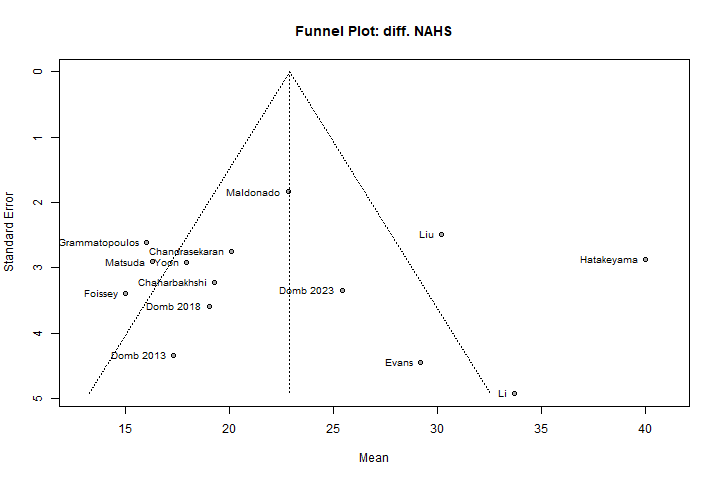

Supplement: Supplementary file 31 — Figure S31. Funnel plot change in NAHS. NAHS: Non‐Arthritic Hip Score. [file JEO2-12-e70311-s031.png]

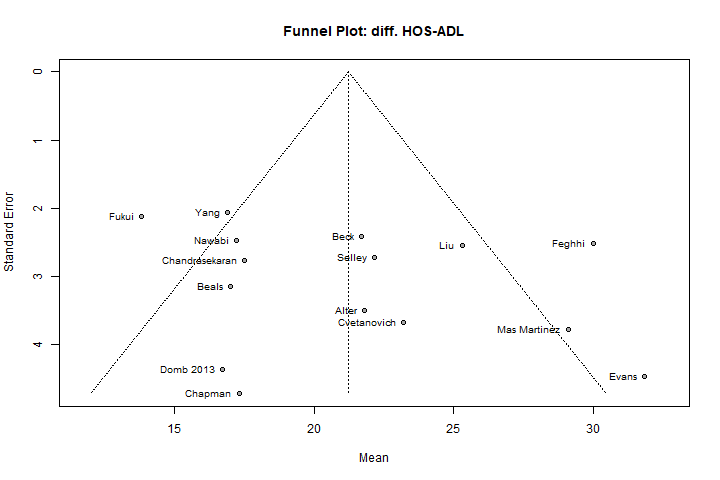

Supplement: Supplementary file 32 — Figure S32. Funnel plot change in HOS‐ADL. HOS‐ADL: Hip Outcome Score ‐ Activities of Daily Living. [file JEO2-12-e70311-s023.png]

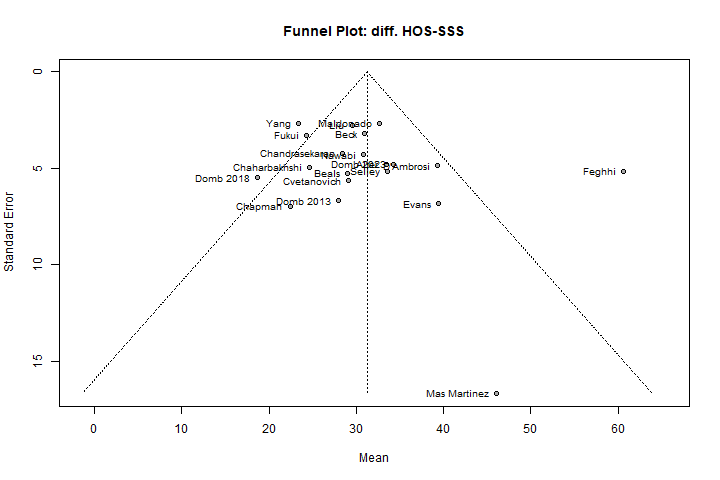

Supplement: Supplementary file 33 — Figure S33. Funnel plot change in HOS‐SSS. HOS‐SSS: Hip Outcome Score – Sport Subscale. [file JEO2-12-e70311-s003.png]

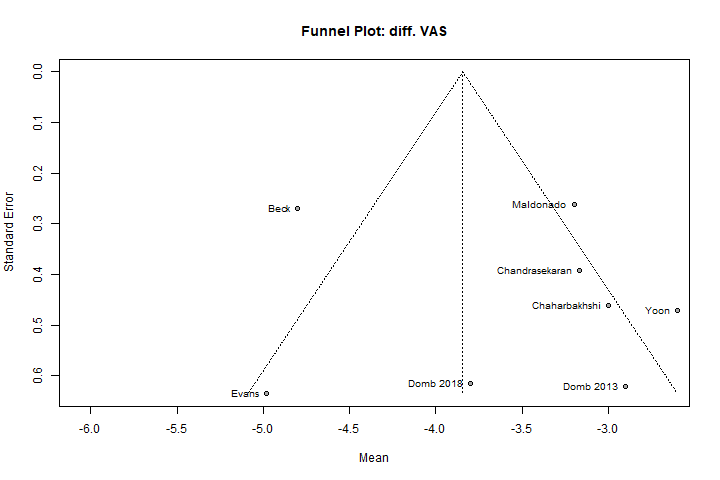

Supplement: Supplementary file 34 — Figure S34. Funnel plot change in VAS. VAS: Visual Analog Scale. [file JEO2-12-e70311-s016.png]

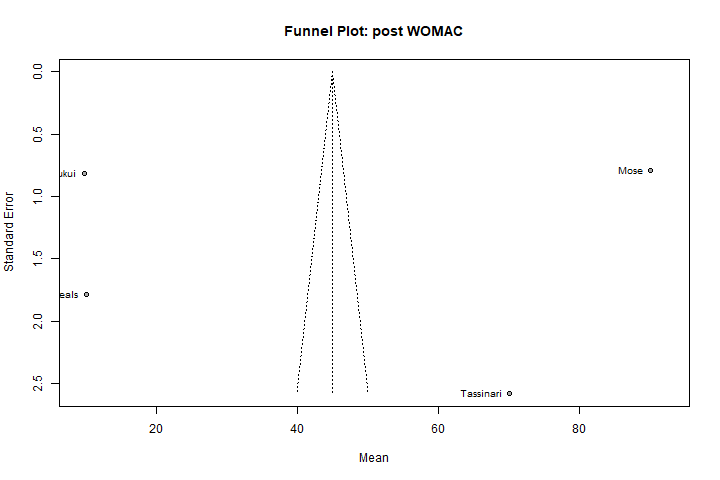

Supplement: Supplementary file 35 — Figure S35. Funnel plot post‐operative WOMAC. WOMAC: Western Ontario and McMaster Universities Osteoarthritis Index. [file JEO2-12-e70311-s024.png]

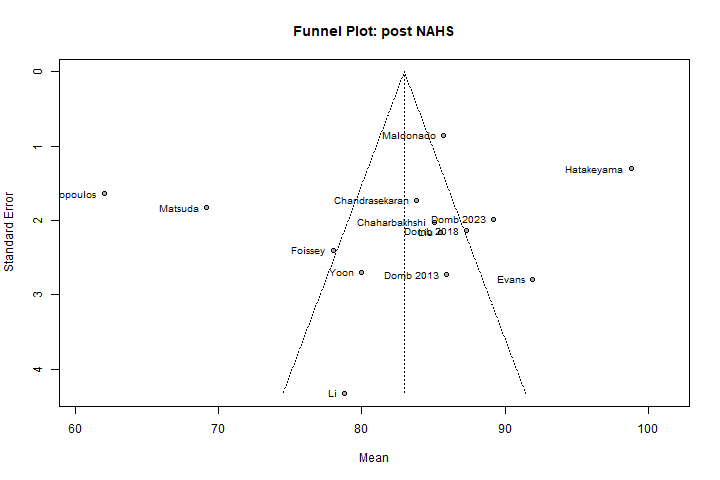

Supplement: Supplementary file 36 — Figure S36. Funnel plot post‐operative NAHS. NAHS: Non‐Arthritic Hip Score. [file JEO2-12-e70311-s034.png]

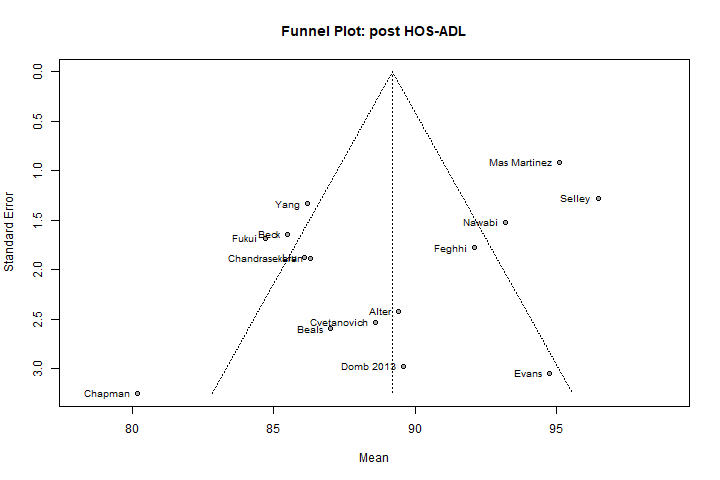

Supplement: Supplementary file 37 — Figure S37. Funnel plot post‐operative HOS‐ADL. HOS‐ADL: Hip Outcome Score ‐ Activities of Daily Living. [file JEO2-12-e70311-s006.png]

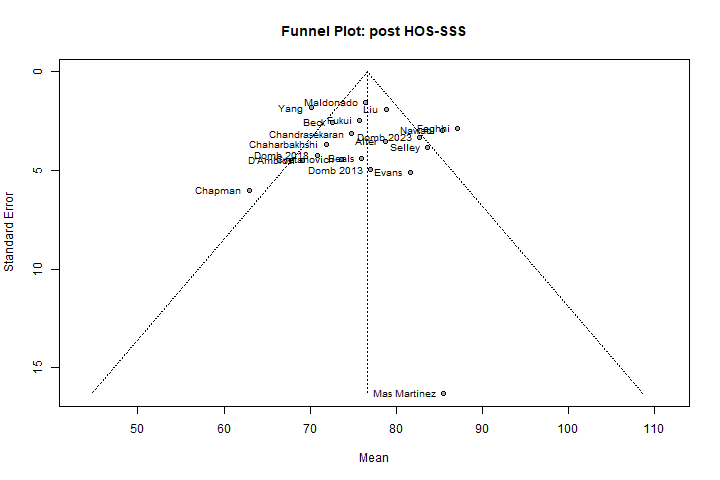

Supplement: Supplementary file 38 — Figure S38. Funnel plot post‐operative HOS‐SSS. HOS‐SSS: Hip Outcome Score – Sport Subscale. [file JEO2-12-e70311-s011.png]

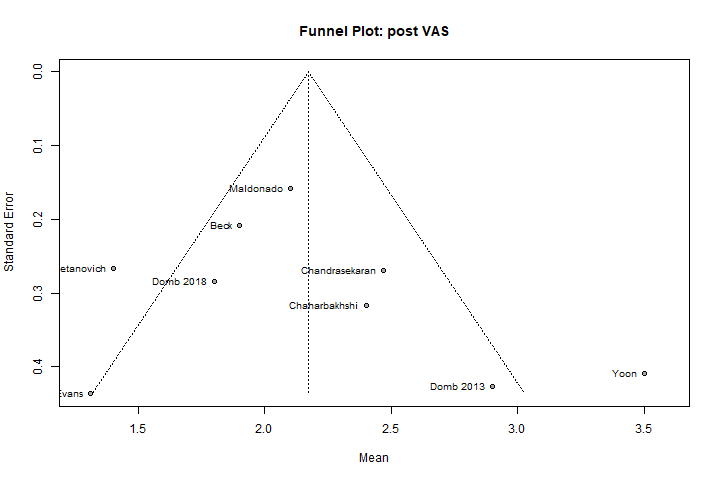

Supplement: Supplementary file 39 — Figure S39. Funnel plot change in VAS. VAS: Visual Analog Scale. [file JEO2-12-e70311-s001.png]

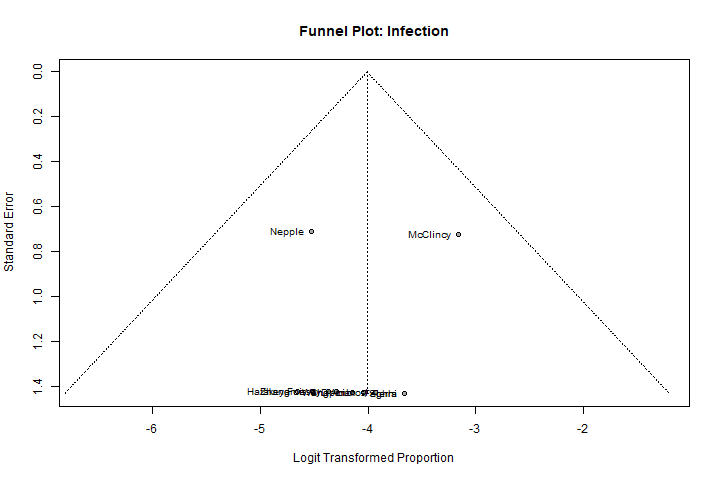

Supplement: Supplementary file 40 — Figure S40. Funnel plot Infection. [file JEO2-12-e70311-s042.png]

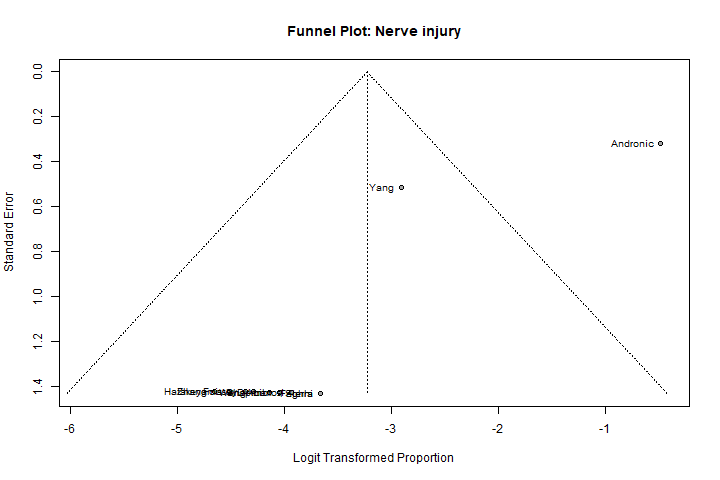

Supplement: Supplementary file 41 — Figure S41. Funnel plot Nerve injury. [file JEO2-12-e70311-s018.png]

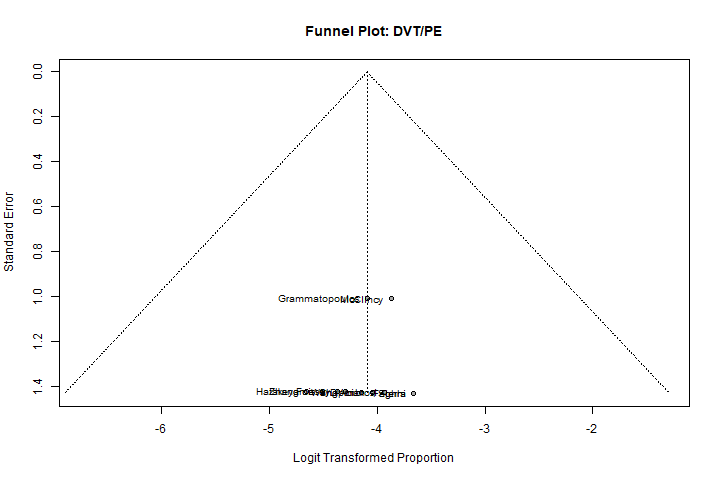

Supplement: Supplementary file 42 — Figure S42. Funnel plot DVT/PE. DVT: deep vein thrombosis; PE: pulmonary embolism. [file JEO2-12-e70311-s039.png]

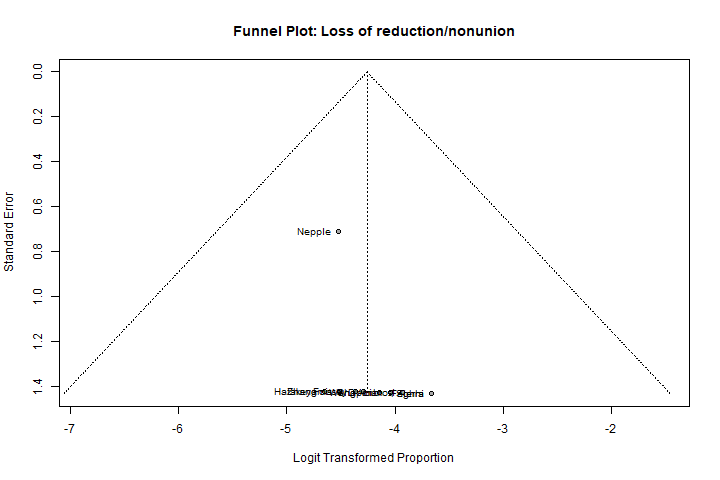

Supplement: Supplementary file 43 — Figure S43. Funnel plot Loss reduction/Nonunion. [file JEO2-12-e70311-s021.png]

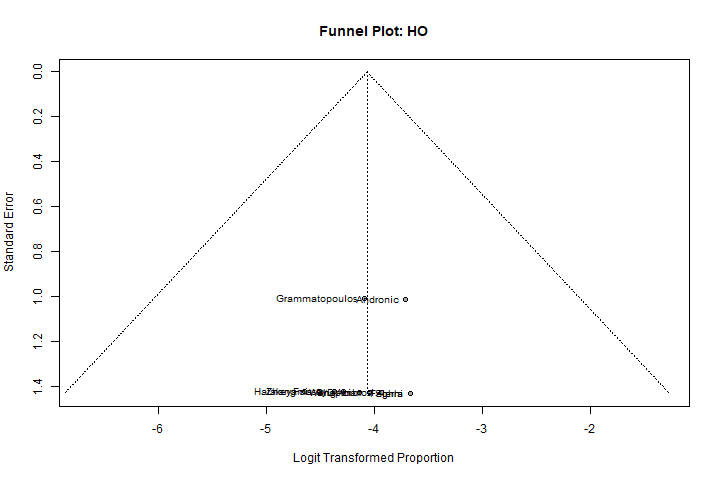

Supplement: Supplementary file 44 — Figure S44. Funnel plot Heterotopic ossification. [file JEO2-12-e70311-s038.png]
